# Growth‐regulating factor 5 (GRF5)‐mediated gene regulatory network promotes leaf growth and expansion in poplar

**DOI:** 10.1111/nph.17179

**Published:** 2021-02-14

**Authors:** Wenqi Wu, Jiang Li, Qiao Wang, Kaiwen Lv, Kang Du, Wenli Zhang, Quanzi Li, Xiangyang Kang, Hairong Wei

**Affiliations:** ^1^ Beijing Advanced Innovation Center for Tree Breeding by Molecular Design Beijing Forestry University Beijing 100083 China; ^2^ State Key Laboratory of Tree Genetics and Breeding Chinese Academy of Forestry Beijing 100091 China; ^3^ State Key Laboratory of Tree Genetics and Breeding Northeast Forestry University Harbin Heilongjiang 150040 China; ^4^ State Key Laboratory for Crop Genetics and Germplasm Enhancement Nanjing Agricultural University Nanjing Jiangsu 210095 China; ^5^ College of Forest Resources and Environmental Science Michigan Technological University Houghton MI 49931 USA

**Keywords:** cytokinin, gene regulatory network, growth‐regulating factor, leaf growth, leaf size, *Populus*, triploid

## Abstract

- Although polyploid plants have larger leaves than their diploid counterparts, the molecular mechanisms underlying this difference (or trait) remain elusive.
- Differentially expressed genes (DEGs) between triploid and full‐sib diploid poplar trees were identified from two transcriptomic data sets followed by a gene association study among DEGs to identify key leaf growth regulators. Yeast one‐hybrid system, electrophoretic mobility shift assay, and dual‐luciferase assay were employed to substantiate that PpnGRF5‐1 directly regulated *PpnCKX1*. The interactions between PpnGRF5‐1 and growth‐regulating factor (GRF)‐interacting factors (GIFs) were experimentally validated and a multilayered hierarchical regulatory network (ML‐hGRN)‐mediated by *PpnGRF5‐1* was constructed with top‐down graphic Gaussian model (GGM) algorithm by combining RNA‐sequencing data from its overexpression lines and DAP‐sequencing data.
- PpnGRF5‐1 is a negative regulator of *PpnCKX1*. Overexpression of *PpnGRF5‐1* in diploid transgenic lines resulted in larger leaves resembling those of triploids, and significantly increased zeatin and isopentenyladenine in the apical buds and third leaves. PpnGRF5‐1 also interacted with GIFs to increase its regulatory diversity and capacity. An ML‐hGRN‐mediated by PpnGRF5‐1 was obtained and could largely elucidate larger leaves.
- PpnGRF5‐1 and the ML‐hGRN‐mediated by PpnGRF5‐1 were underlying the leaf growth and development.

## Introduction

The majority (75%) of polyploids are allopolyploids (Grant, 1981; Brochmann *et al*., 2004). The prevalence of polyploidy in flowering plants implies some evolutionary advantages over diploids (Wood *et al*., 2009). Triploids usually manifest characteristics such as larger diameters, higher heights, larger leaf areas, and higher biomass accumulation (Guo, 2004; Auger *et al*., 2005). Recent studies on several species have shown that the growth advantage of leaves in polyploids is more salient than any other trait, for example, poplar triploids (Liao *et al*., 2016) and tetraploids (Xu *et al*., 2016), birch tetraploids (Mu *et al*., 2012), *Eucommia ulmoides* triploids (Li *et al*., 2019) and tetraploids (Tokumoto *et al*., 2016) and *Eucalyptus* triploids (Yang *et al*., 2018) and tetraploids (Fernando *et al*., 2019). The fact that both triploids and tetraploids display much larger leaves than their comparable (e.g. full‐sib) diploids implies that the ploidy is the primary cause of bigger leaf areas in polyploids. In *Arabidopsis thaliana*, both leaf area and cell size in tetraploids are also consistently larger than diploids (Ni *et al*., 2009).

*Growth‐Regulating Factors* (*GRFs*) belong to a small plant‐specific transcription factor (TF) family. GRF proteins generally harbor conserved QLQ and WRC domains in their N‐terminal regions (Kim & Kende, 2004). The QLQ domain interacts with the SHN domain of GRF‐INTERACTING FACTOR 1 (GIF1) protein, and GIF1 is associated with plant SWI/SNF chromatin remodeling complexes (Horiguchi *et al*., 2005; Vercruyssen *et al*., 2014). The WRC domain contains a functional nuclear localization signal and a DNA‐binding motif (van der Knaap *et al*., 2000; Kim *et al*., 2003). The C‐terminal regions of GRF proteins diverge in sequence from each other but have common features that are reminiscent of TFs (Kim & Kende, 2004).

GRFs function in regulating leaf (Kim *et al*., 2003) and stem (van der Knaap *et al*., 2000) development, shoot apical meristem development (Kim & Lee, 2006), leaf primordia formation (Horiguchi *et al*., 2005), and leaf size and longevity (Debernardi *et al*., 2014). As stated earlier, GRFs form a transcriptional complex with GIFs to confer meristematic potential during organogenesis (Kim, 2019). In addition, the transcripts of most *GRFs* are often targeted by *miR396* for degradation (Liu *et al*., 2009). Overexpression of *GRFs* promotes the expression of cell cycle genes and auxin response genes, resulting in increased cell numbers and expansion of leaves (Omidbakhshfard *et al*., 2015; Piya *et al*., 2020).

TFs are important components in regulatory cascades during plant development. Identification of important high hierarchical regulators, their direct and indirect downstream target genes, as well as some co‐regulators at the same level, is critical to understand phenotypical and complex trait formation. This goal can be achieved by building a multilayered hierarchical gene regulatory network (ML‐hGRN) starting from a TF after perturbating it (Wei, 2019). Our previously developed top‐down graphic Gaussian model (GGM) enables construction of a ML‐hGRN‐mediated by a TF (Lin *et al*., 2013; Wei, 2019). The method has been used to construct GRNs that control wood formation in poplar (Chen *et al*., 2019) and that governs the adventitious root formation in poplar (Wei *et al*., 2020).

In this study, we attempted to dissect the molecular mechanisms underlying larger leaf size in poplar triploids with the full‐sib diploids as a comparison. The expression levels of *PpnGRF5‐1* were particularly high in the apical buds and young leaves of triploids compared to those in the full‐sib diploids. PpnGRF5‐1 inhibited *PpnCKX1* expression, resulting in the accumulation of cytokinins to promote the meristematic potential of the proliferative and formative cells during leaf development. *PpnGRF5‐1* overexpression transgenic lines in diploids had larger leaves resembling those of triploids. We generated RNA‐sequencing (RNA‐seq) data from these transgenic lines and then reconstructed PpnGRF5‐1‐mediated ML‐hGRN by integration of the PpnGRF5‐1 and target gene relationships acquired from DNA affinity purification sequencing (DAP‐seq) experiment. The fact that the larger leaves resulting from *PpnGRF5‐1* overexpression in transgenic diploids and significantly high expression of *PpnGRF5‐1* in triploids implies that PpnGRF5‐1‐mediated ML‐hGRN contributed to leaf growth and development, resulting in much larger leaves.

## Materials and Methods

### Plant materials propagation

The allotriploid populations (2*n* = 3*x* = 57) of *Populus* section *Tacamahaca* (*P*. *pseudo‐simonii* × *P*. *nigra* ‘Zheyin 3#’, Ppn) and full‐sib diploid (2*n* = 2*x* = 38) population were generated in our previous study (Cheng *et al*., 2015). We randomly drew 30 genotypes from these populations, and vegetatively propagated them through cuttings for leaf phenotypic study and characterization of tissue‐specific gene expression. The stem cuttings of 15 cm long were planted in peat soil in a glasshouse (16 h : 8 h, light : dark, 22°C : 25°C).

### Microscopic observations

For histological analysis of cells, mature leaves were fixed overnight in FAA (formalin : acetic acid : ethanol, 1 : 1 : 18), and cleaned with chloral solution (200 g chloral hydrate, 20 g glycerol, and 50 ml double‐distilled water (ddH_2_O)) as described (Horiguchi *et al*., 2005). Palisade leaf cells were observed by differential interference contrast microscopy (BX61; Olympus, Tokyo, Japan). Palisade cells in the center of the leaf blade, between the midvein and the leaf margin, were analyzed. Cell area was determined by measuring 40 palisade cells per leaf. The total number of palisade cells in the subepidermal layer was calculated by dividing the leaf area by the palisade cell area.

### RNA‐seq experiment and data analysis for identifying candidate genes regulating leaf growth in triploid

We generated a transcriptome data set (available at Genome Sequence Archive with an accession no. CRA003631) of the apical buds, third and fifth leaves of the same triploid and the full‐sib diploid population (4‐month‐old) as mentioned earlier. There were two biological replicates, each was mixed from five plants. Furthermore, 2 μg RNA per sample was used for library preparation with RNA‐NEBNext Ultra RNA Library Prep Kit. RNA‐seq was performed by Novogene Co. Ltd (Beijing, China) using Illumina HiSeq 4000 platform, and approximately 6 GB of 150‐bp paired‐end reads were generated from each library. The clean reads mapped to *P. trichocarpa* genome 4.1 using TopHat2 (Kim *et al*., 2013), and raw count of each gene was obtained with Bedtools (Quinlan & Hall, 2010). We then used Pop’s pipe (Li *et al*., 2014) to identify differential expressed genes (DEGs) and then performed gene association study among DEGs using Spearman rank correlation (Kumari *et al*., 2012) to identify candidate genes regulating leaf growth. The cut‐off threshold for DEGs was set to the false discovery rate < 0.05.

The second leaf transcriptomic data set (available at Genome Sequence Archive with an accession no. CRA003633) generated in our earlier study (Du *et al*., 2020) was used to identify candidate genes regulating leaf growth. The data set was generated from the fifth, 10th and 25th leaves of the same triploid and the full‐sib diploid population. The clean reads were aligned to *P. trichocarpa* genome 4.1 using Burrows Wheeler Aligner (BWA) (Li & Durbin, 2010) and raw read count of each gene using HTseq‐count (Anders *et al*., 2015). The identification of DEGs and gene association study used the same methods as aforementioned.

### Tissue‐specificity of gene expression

A total of 11 tissues included the apical buds with unexpanded leaflets, the first, third, fifth, and seventh leaves, phloem, the internode between the apical bud and the first opened leaf (internode 1), the internode between the first and second leaves (internode 2), root tips (root 1), the long white roots (without side roots) (root 2), and small side roots (root 3) were harvested from 3‐month‐old poplars for extracting total RNA with TRIzol reagent according to the manufacturer’s instructions (cat. no. 15596026; Thermo Fisher, Waltham, MA, USA). The more detailed information for complementary DNA (cDNA) synthesis and reverse transcriptase quantitative polymerase chain reaction (RT‐qPCR) was provided in Methods S1–S10 of the Supporting Information. Three independent biological replicates were used in the analysis. Primers are listed in the Supporting Information, Table S1.

### Construction of plasmid vectors and gene transformation

The coding sequence of *PpnGRF5‐1* was amplified from the cDNA of allotriploid and inserted into the *pBI121‐eGFP* vector containing the CaMV35S promoter (35S) to generate the *35S::PpnGRF5‐1‐eGFP‐nos* construct. Primers are listed in Table S1. *Agrobacterium tumefaciens*‐mediated transformation of 84K clone (*P*. *alba* × *P*. *glandulosa*, Pag) that is transformation‐amenable for gene transformation following the protocol as described earlier (Liu *et al*., 2014). We confirmed positive transgenic lines by PCR, and then screened them to obtain transgenic lines with variable expression levels of *PpnGRF5‐1* based on RT‐qPCR (low, medium, high), and propagated them for leaf phenotypic studies, RNA‐seq experiment, and cytokinin measurement.

### RNA‐seq experiment of transgenic lines and data analysis

The apical buds of *PpnGRF5‐1* overexpression lines were harvested. For each line, the apical buds of five plants with similar growth were selected and mixed. The 15 apical buds of wild‐type plants were collected and divided into three groups as controls. RNA extraction, library construction and sequencing were done at Annoroad Corp. (Beijing, China) using the same protocols and approximately 4GB of 150‐bp paired‐end reads were generated from each library. The clean reads were mapped to the 84K polar genome using TopHat2 (Kim *et al*., 2013). Raw reads that mapped to annotated genes were counted using HTseq‐count (Anders *et al*., 2015). DEGs between *PpnGRF5‐1* overexpression lines and wild‐type (WT) were identified using the Pop’s Pipes pipeline (Li *et al*., 2014). These overexpression lines were classified into three groups with high, medium, and low expression levels of *PpnGRF5‐1* for identifying DEGs separately by comparing to a group of WT. The cut‐off threshold for DEGs was set to false discovery rate < 0.05.

### Gene ontology (GO) enrichment analysis

The DEGs were used for gene ontology (GO) analysis using AmiGO's Term Enrichment tool (http://amigo.geneontology.org/). The R module clusterprofiler available at Bioconductor (http://bioconductor.org) was used to identify the enriched GO terms associated with a DEG list via hypergeometric probability. We applied a multiple testing correction using the Benjamini & Hochberg (1995) false discovery rate method. GO terms with a corrected *P*‐value < 0.01 were significantly enriched. The emapplot function in clusterprofiler was used to generate the network in which the different gene sets (each was represented by a node) with at least one mutually overlapping gene were connected with each other.

### Construction of gene regulatory network (GRN)

We used the top‐down GGM algorithm (Lin *et al*., 2013; Wei, 2019) to construct an ML‐hGRN‐mediated by PpnGRF5‐1. The transcriptomic data generated from specifically screened overexpression transgenic lines with low, medium, and high levels of *PpnGRF5‐1* were used to identify DEGs. The top‐down GGM algorithm was employed to build an ML‐hGRN in two steps that were described in great detail in two of our early publications (Lin *et al*., 2013; Wei, 2019): (1) identification of PpnGRF5‐1‐responsive genes from the DEGs using Fisher’s exact test and a probability‐based method; (2) identification of direct target genes from PpnGRF5‐1‐responsive DEGs that had causal relationships with PpnGRF5‐1 by evaluating triple gene blocks, *PpnGRF5‐1* (z) and two of the combined PpnGRF5‐1‐responsive DEGs, *x* and *y*. If the *PpnGRF5‐1* significantly interfered with the two responsive genes, namely $d=r_{\mathrm{xy}}‐r_{xy|z}$ is significant after being tested with the multivariate delta method (MacKinnon *et al*., 2002), then *PpnGRF5‐1* was considered to control the two genes and their regulatory relationships (edges) were recorded. When PpnGRF5‐1 and all combinations of all responsive genes were evaluated, the interference frequency between *PpnGRF5‐1* and each responsive candidate target gene was calculated. Then, the *PpnGRF5‐1*‐responsive candidate target genes having the highest frequency and *PpnGRF5‐1*‐binding motif were retained in this middle layer (directly targets). The next layer (bottom) was generated in top‐down fashion only from each TF present in the middle layer by recursively calling top‐down GGM algorithm. Additional details can be found in Methods S1.

All other routinely used methods including RNA isolation, RT‐PCR, RT‐qPCR (Methods S2), transcriptional activation analysis (Methods S3), yeast one‐hybrid (Y1H) assays (Methods S4), electrophoretic mobility shift assay (Methods S5), DAP‐seq (Methods S6), dual‐luciferase (LUC) assay (Methods S7), yeast two‐hybrid (Y2H) assays (Methods S8), pull‐down assay (Methods S9), and split LUC complementation assay (Methods S10), used in this study are described in the Supporting Information.

## Results

### Bigger leaf sizes in triploid poplar resulted from complex coordination of cell division and expansion

**Fig. 1:**
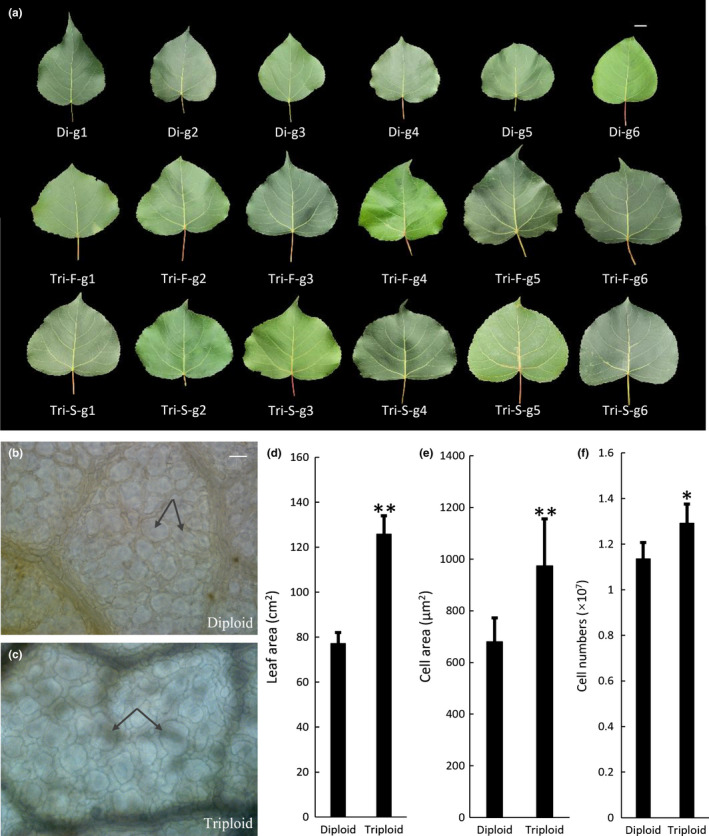
Phenotypic analysis of triploid and full‐sib diploid poplars. (a). The fifth leaves were harvested from randomly selected six genotypes of triploid populations and their full‐sib diploid population. Bar, 2 cm. Di, diploid; Tri, triploid; F, first division restitution (FDR) gametes; S, second division restitution (SDR) gametes; g1, genotype 1. (b, c) Paradermal view of palisade cells in the fifth leaves from 3‐month‐old diploid and triploid poplars (eight leaves were examined). Bars, 10 μm. (d–f) Leaf area, cell area, and calculated cell numbers of fifth leaves from 3‐month‐old diploid and triploid poplars (30 leaves were measured). *, *P* < 0.05; **, *P* < 0.01 (determined by Student’s *t*‐test). Values represent the mean ± SD (*n* = 30).

It has previously been reported that the triploid poplar trees have noticeable vegetative growth advantages compared to the full‐sib diploid trees (the progenies of the same parents of triploids). The most striking advantages are greater height, larger breast diameter, larger leaf area, augmented stress resistance, and lower fertility (Liao *et al*., 2016). One of the most stable and salient growth advantages of triploid poplar trees was that all of them have much larger leaves (Fig. 1a,d). We found that the average size of leaves (randomly collected 30 genotypes, fifth leaf blade) (Supporting Information, Fig. S1) from triploid poplar were approximately 63.02% larger, respectively, compared to leaves from full‐sib diploid poplar. Since it is not clear whether cell division, cell expansion, or both contribute to faster leaf growth in triploids, we studied the paradermal view of palisade cells in at least eight samples of the fifth leaf blades from apical buds. Palisade cells were significantly enlarged in triploids compared to diploids (Fig. 1b,c,e); the average area of palisade cells in triploids was 43% larger than in diploids (Fig. 1e). Based on the average leaf area (Fig. 1d) and cell sizes, we calculated the average number of cells per leaf which was 1.22 × 10^7^ and 1.14 × 10^7^ in triploids and diploids, respectively (Fig. 1f). Therefore, the enlarged leaves of triploid poplar result from augmented cell division and cell expansion.

### Identification of candidate genes that control leaf growth in triploid poplar

We obtained transcriptomic data from triploid population of first division restitution (FDR) gametes and their full‐sib diploid population. We performed gene association studies using two transcriptomic data sets from the triploids and their full‐sib diploids, and found that *PpnGRF5* and cytokinin oxidase/dehydrogenase 1 (*PpnCKX1*), which were upregulated and downregulated in the leaves of triploids, respectively, had a negative correlation in both data sets (Fig. S2). Based on this, we hypothesized that *PpnGRF5* might specifically target *PpnCKX* for regulation, modulating leaf growth in triploids. We therefore employed a series of molecular and biochemical means to substantiate the regulation of *PpnGRF5* on *PpnCKX1* in this study.

### Tissue‐specificity of *PpnGRF5* in triploid poplar

**Fig. 2:**
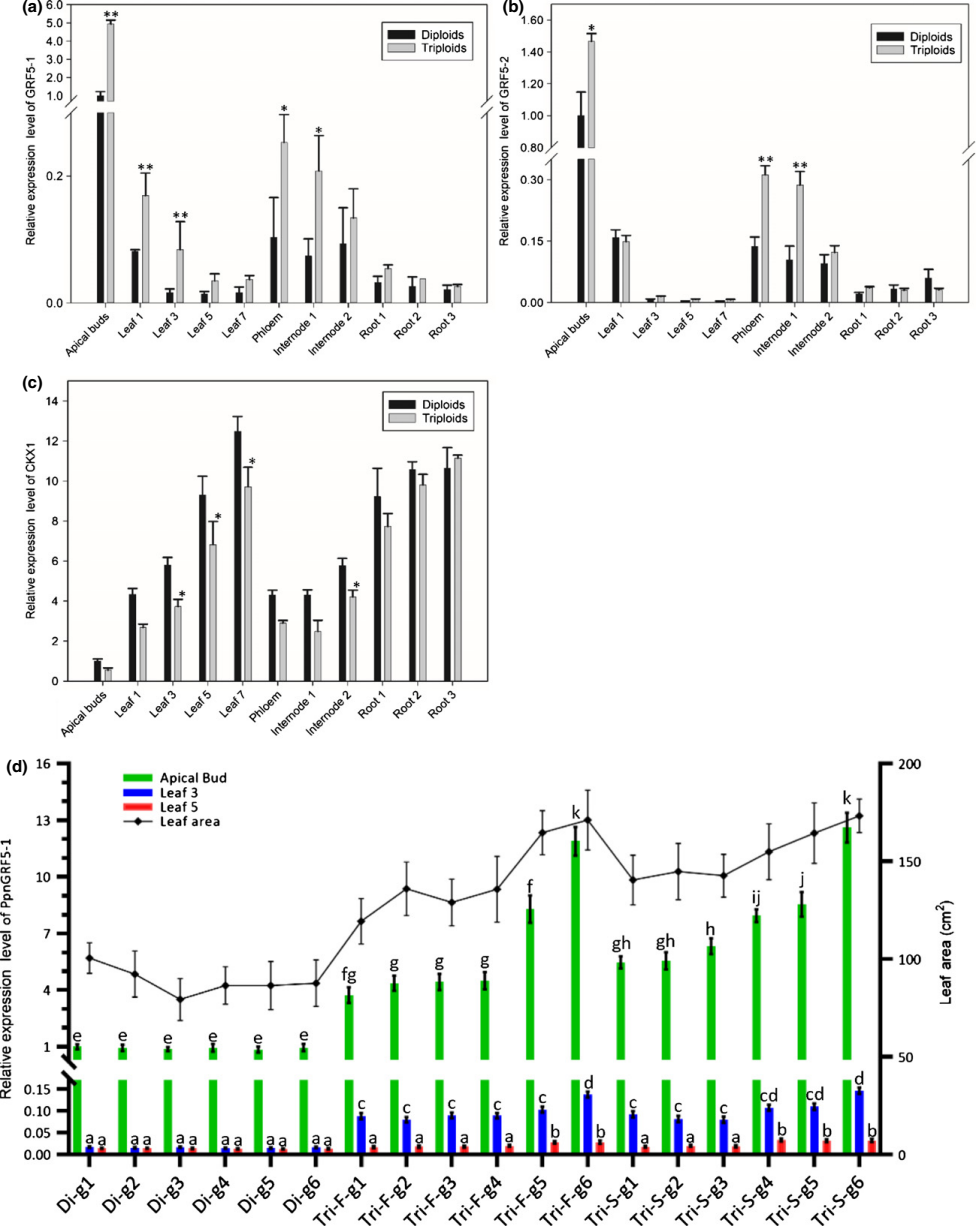
Expression analyses of *PpnGRF5* and *PpnCKX1* in triploid poplars. (a, b, c) Tissue‐specific expression patterns of *PpnGRF5‐1*, *PpnGRF5‐2*, and *CKX1* genes relative to *ACTIN* in vegetative tissues of 3‐month‐old triploid and diploid poplar plants. *, *P* < 0.05; **, *P* < 0.01 (determined by Student’s *t*‐test). (d) Bar chart: expression level of PpnGRF5‐1 in apical buds, the third leaves, and the fifth leaves in six different genotypes of 4‐month‐old triploid and full‐sib diploid poplars. Different letters denote statistically significant differences resulting from Tukey’s range test following two‐way ANOVA. Values represent the mean ± SD (*n* = 3). Line plot: leaf area of the fifth leaves of the different genotypes of diploid and triploid poplars. Values represent the mean ± SD (*n* = 10). Di, diploid; Tri, triploid; F, first division restitution (FDR) gametes; S, second division restitution (SDR) gametes; g1, genotype 1.

There are two *PpnGRF5* genes, *PpnGRF5‐1* (*Potri.013G077500*) and *PpnGRF5‐2* (*Potri.018G065400*), expressed in apical buds in poplar triploids (Fig. S3). To study their tissue‐specific expression patterns, we collected 11 different tissues from 3‐month‐old triploids and full‐sib diploids and then employed RT‐qPCR to quantify *PpnGRF5* and *PpnCKX1* genes. In both triploids and diploids, *PpnGRF5* genes had roughly similar tissue‐specific expression patterns. First, both *PpnGRF5* genes were more highly expressed in the apical buds than any other tissue (Fig. 2a,b). Second, the expression levels of both *PpnGRF5* genes decreased as the leaf number/age increased (Fig. 2a,b). Third, expression of both *PpnGRF5* genes was relatively low and at nearly the same level in each of the different types of roots. Therefore, these two genes may function primarily in apical buds and young leaves (Fig. 2a,b).

As shown in Fig. 2a,b, both *PpnGRF5* genes were differentially expressed in some tissues in triploids compared to diploids; in all tissues where significant differences were found, there was increased expression in triploids compared to diploids. Most strikingly, the expression of the *PpnGRF5‐1* gene in the apical buds of triploid poplar was almost five times higher than in the diploid, but *PpnGRF5‐1* expression dropped dramatically in the fifth leaves compared with apical buds. In internodes 1 and 2, *PpnGRF5‐1* expression in triploids was at least twice that in diploids (Fig. 2a). In the apical buds and internode 1, expression of the *PpnGRF5‐2* gene was significantly higher in triploids than in diploids, though less than two times (Fig. 2b). Such a significant increase in *PpnGRF5* gene expression in the apical buds and first leaves of triploids indicates that the two *PpnGRF5* genes play a role in promoting leaf growth and development in triploids. Tissue‐specific expression patterns indicated that *PpnGRF5‐1* expression was higher in various tissues, especially apical buds and young leaves, than *PpnGRF5‐2*. This suggested that *PpnGRF5‐1* may play a more important role than *PpnGRF5‐2* in the meristematic potential of proliferative and formative cells during leaf growth. Therefore, our study hereafter primarily focused on *PpnGRF5‐1*.

To investigate if the size of mature leaf in triploids have a correlation with the elevated *PpnGRF5‐1* expression, we measured its expression and leaf areas of the six genotypes randomly drawn from each of three populations: one full‐sib diploids and two (FDR and second division restitution (SDR) gametes) triploids. The expression levels of *PpnGRF5‐1* in apical buds, the third and fifth leaves of each genotype were measured by RT‐qPCR (Fig. 2d). The areas of the fifth leaves of different genotypes were measured and added to Fig. 2d. A discernible correlation was observed between the areas of the fifth fully expanded leaf and the expression levels of *PpnGRF5‐1* of any of all three leaf positions especially apical buds.

### PpnGRF5‐1 interacts with poplar PpnGIFs

**Fig. 3:**
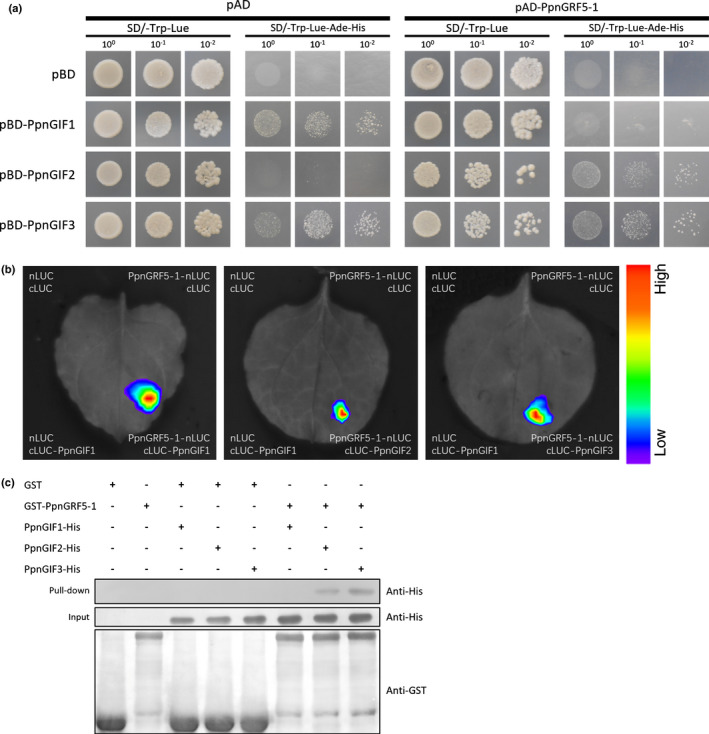
PpnGRF5‐1 interacts with PpnGIFs. (a) Yeast cells of co‐transformants of PpnGRF5‐1 and GIFs grown on SD/‐Trp‐Leu and SD/‐Trp‐Leu‐His‐Ade medium at 30°C for 3 d. PpnGRF5‐1 was fused to transcription activation domain (AD), and GIFs were fused to DNA‐binding domain (BD). pAD and pBD are negative controls. (b) Split‐luciferase (LUC) complementation assay reveals the interaction between PpnGRF5‐1 and GIFs. PpnGRF5‐1 was fused to the N‐terminal portions of LUC (nLUC), and GIFs were fused to the C‐terminal portion of LUC (cLUC). *Agrobacteria* carrying different plasmids as indicated were co‐expressed in *Nicotiana benthamiana*. Representative images of *N. benthamiana* leaves 48 h after infiltration are shown. Color scale represents LUC activity. The experiment was repeated three times with similar results. (c) *In vitro* pull‐down assays assessing physical interactions between PpnGRF5‐1 and GIFs. GST‐PpnGRF5‐1 was incubated in binding buffer containing glutathione‐agarose beads with or without PpnGIFs‐6 × His, and agarose beads were washed five times and eluted. Lysis of *Escherichia coli* (Input) and eluted proteins (Pull down) from beads was immublotted using anti‐HIS and anti‐GST antibodies.

We cloned *PpnGRF5‐1* from triploids and confirmed that it contains 1023 bp nucleic acids, corresponding to 340 amino acids. The nucleic and amino acid sequences were 98.83% and 98.82% identical to their respective counterparts in *P. trichocarpa*. The sequence features and transactivation activity test showed that PpnGRF5‐1, which harbors one QLQ, one WRC domain and conserved TQL motif, functioned as a transcriptional factor and located in the nucleus (Figs S4, S5). GRF proteins have been reported to interact with a transcriptional cofactor, GIF, and form a functional transcriptional complex that may be essential for regulating leaf organogenesis (Kim, 2019). To investigate if PpnGRF5‐1 indeed interacts with PpnGIFs in poplar, we cloned three poplar *PpnGIF* genes, *PpnGIF1*, *PpnGIF2*, and *PpnGIF3*, from the triploid poplar tree. These three *GIFs* have 99.55%, 99.68%, and 99.55% similarity to their counterparts, *Potri.013G043700* (*PtrGIF1*), *Potri.014G103900* (*PtrGIF2*), and *Potri.019G013100* (*PtrGIF3*), in *P. trichocarpa*, respectively. We tested the physical interactions between PpnGRF5‐1 and each of these three PpnGIFs using a Y2H system. However, because strong autoactivation was observed for PpnGRF5‐1 when fused to the Gal4 DNA binding domain, the assay was performed only in one direction. The interaction between PpnGRF5‐1 and PpnGIF2 was explicitly evidenced by gradient dilution (Fig. 3a). Unfortunately, PpnGIF1 and PpnGIF3 autoactivated the reporter genes even in the absence of PpnGRF5‐1 protein, and the autoactivation of GIF1 vanished when co‐transformed with PpnGRF5‐1 for some unknown reason (e.g. abnormal protein folding/interaction) (Fig. 3a). Next, we employed a split LUC complementation assay to test PpnGRF5‐1 and PpnGIFs interactions. In our experiment, we injected *35S::PpnGRF5‐1‐nLUC* and *35S::cLUC‐PpnGIFs* into *Nicotiana benthamiana* leaf epidermal cells. Two days later, only co‐expression of PpnGRF5‐1 and PpnGIFs at the same sites resulted in the reconstitution of LUC and detectable fluorescence signal (Fig. 3b). No bioluminescence was observed when either *35S::PpnGRF5‐1‐nLUC* or *35S::cLUC‐PpnGIFs* was co‐expressed with *35S::cLUC* or *35S::nLUC*, respectively (Fig. 3b). Furthermore, the interaction was also verified by a third method, immuno‐pull‐down, using purified recombinant GST‐PpnGRF5‐1 and PpnGIFs‐His_6_ proteins. In this experiment, immobilized GST‐PpnGRF5‐1, but not GST (glutathione‐*S*‐transferase) alone, bound to PpnGIF2‐His_6_ and PpnGIF3‐His_6_ (Fig. 3c). Based on the results of the three assays described earlier, we concluded that PpnGRF5‐1 and PpnGIF2 interacted in all three assays, PpnGRF5‐1 interacted with PpnGIF3 in two assays, and PpnGRF5‐1 interacted with PpnGIF1 only in one assay. Multiple interacting partners indicate that diverse complexes may be formed to regulate a multitude of target genes.

### *PpnGRF5‐1* significantly enlarged leaf size in *PpnGRF5‐1* overexpression transgenic lines by enhancing both cell division and cell expansion

**Fig. 4:**
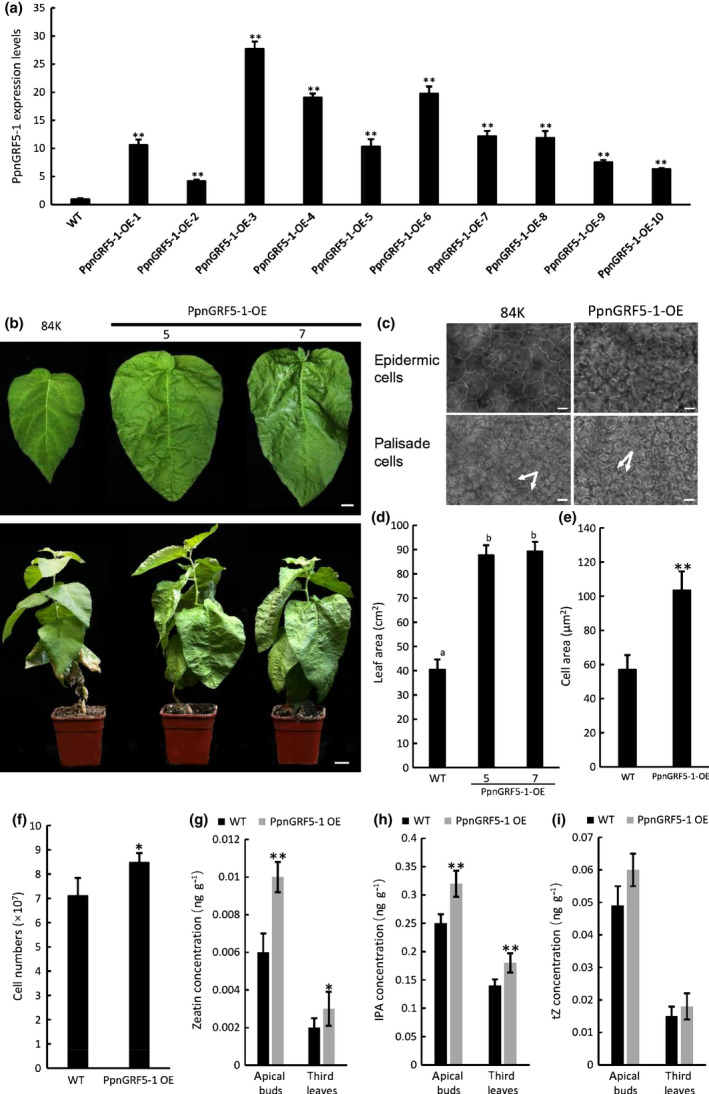
Phenotypic and cytokinin content analysis of *PpnGRF5‐1* overexpression transgenic 84K lines. (a) Expression levels of *PpnGRF5‐1* in the apical buds (including unexpended leaflets) of *PpnGRF5‐1* overexpression (OE) lines. Values represent the mean ± SD (*n* = 5). (b) The fifth leaves (upper panel) of 3‐month‐old *PpnGRF5‐1* overexpression and 84K wild‐type (WT) poplar trees and whole trees (lower panel) by tissue culture grown in soil in pots. Bars: 1 cm in leaves, 2 cm in trees. (c) Paradermal view of epidemic and palisade cells in the fifth leaves from the apical buds of 3‐month‐old *PpnGRF5‐1*‐*OE* poplar trees by tissue culture. Bars, 10 μm. (d–f) Leaf area, cell area, and calculated cell numbers of fifth leaves from 3‐month‐old PpnGRF5‐1 overexpression and WT 84K poplar trees by tissue culture. Different letters denote statistically significant differences resulting from Tukey’s range test following one‐way ANOVA. *, *P* < 0.05; **, *P* < 0.01 (determined by Student’s *t*‐test). Values represent the mean ± SD. (g–i) Cytokinin content, including zeatin (g), isopentenyladenine (IPA) (h), and *trans*‐zeatin (tZ) (i), detected in the apical buds and third leaves in 3‐month‐old *PpnGRF5‐1* overexpression and 84K WT poplar trees from tissue couture. *PpnGRF5‐1*‐*OE* means the mixed leaf samples of *PpnGRF5‐1‐OE* transgenic lines 5 and 7. *, *P* < 0.05; **, *P* < 0.01 (determined by Student’s *t*‐test). Values represent the mean ± SD (*n* = 5).

To test if the larger leaves in triploids were primarily caused by elevated *PpnGRF5‐1* expression, we developed *PpnGRF5‐1* overexpression (*PpnGRF5‐1‐OE*) transgenic lines (*35S::PpnGRF5‐1‐eGFP‐nos* vector) in diploid poplar. We used the 84K poplar clone as our gene transformation system and obtained 10 independent transgenic lines overexpressing *PpnGRF5‐1* (Fig. 4a). Each of these lines exhibited much larger leaves than WT (Figs 4b,d, S6a,b), and the sizes of the fifth leaves of all the 3‐month‐old transgenic lines (diploids) were positively correlated with the expression levels of *PpnGRF5‐1* in the apical bud, the third and the fifth leaves (Fig. S6a,b). The diameters rather than heights of 5‐month‐old *PpnGRF5‐1‐OE* lines (lines 1, 3, 4, 5, 6, 7 and 8) were significantly increased (Table S2). The *PpnGRF5‐1‐OE* #5 and #7, which had moderate overexpression of *PpnGRF5‐1*, were selected for further analyses. The areas of the fifth leaves from *PpnGRF5‐1‐OE* #5 and #7 transgenic lines were 2.16 and 2.2 times larger than that of WT, respectively. *PpnGRF5‐1‐OE* leaves were more wrinkled on the surface than WT leaves, presumably owing to uneven, fast growth (Fig. 4b). To further investigate what caused the leaves in transgenic lines to grow to a larger size than WT leaves, we examined specific cells in the fifth leaf blades; we observed much larger epidermic and palisade cells in *PpnGRF5‐1‐OE* compared to WT leaves (Fig. 4c,e). Moreover, the average number of cells per leaf in *PpnGRF5‐1‐OE* and WT was 8.5 × 10^7^ and 7.4 × 10^7^, respectively (Fig. 4f). Collectively, the positive effect of *PpnGRF5‐1* on leaf growth in *PpnGRF5‐1‐OE* was due to an increase in both leaf cell size and number. These results clearly demonstrate that *PpnGRF5‐1* alone can drive cell division and cell expansion in diploids, indicating that the larger leaves in triploids can be ascribed to the significant upregulation of *PpnGRF5‐1*.

Based on the gene regulatory module obtained earlier, *PpnGRF5‐1* regulates the expression of *PpnCKX1* and should therefore change the concentration of cytokinins in the leaves. To verify this, we measured three types of cytokinins, *trans‐*zeatin (tZ), zeatin, and isopentenyladenine (IPA), in the apical buds and the third leaves of both *PpnGRF5‐1‐OE* and WT plants. In both young and old leaves, zeatin and IPA concentrations were significantly increased in overexpression compared to WT leaves (Fig. 4g–i). Such an obvious increase in cytokinin concentrations in the apical buds and the third leaves of transgenic lines indicates that *PpnGRF5‐1* maybe repressed cytokinin degradation through downregulation of *PpnCKX1*.

### PpnGRF5‐1 govern leaf expansion by regulating PagCKX1

**Fig. 5:**
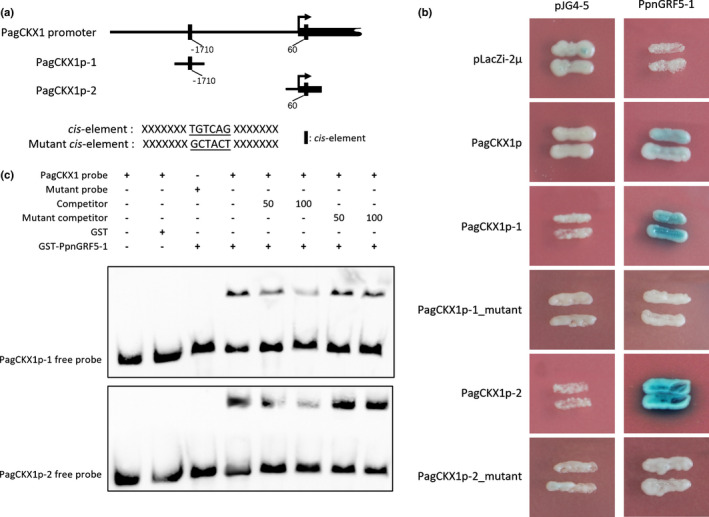
PpnGRF5‐1 binds directly to the *PagCKX1* promoter. (a) The putative PpnGRF binding elements in the *PagCKX1* promoter (upper panel) and mutagenesis of the PpnGRF binding element in the *PagCKX1* promoters (lower panel). The mutant GCTACT was obtained after screening against possible binding variants of PpnGRF5‐1 yielded from DAP‐seq. The absolute value of each number indicates the distance from the start codon. (b) Y1H assay showing the direct binding of PpnGRF5‐1 to the elements in the *PagCKX1* promoter. Blue colonies indicate a strong association of PpnGRF5‐1 with a specific promoter segment. (c) EMSA showing that the GST‐PpnGRF5‐1 recombinant protein binds to biotin‐labeled probes of *PagCKXp‐1* (upper panel) and *PagCKXp‐2* (lower panel). The probes were truncated from *PagCKX1* promoter with putative binding sites (TGTCAG) and mutant probes is a mutated form of probes (Supporting Information Table S1). Here, 50 and 100 unlabeled probes and probe mutants were used in the competition experiment.

The transcriptomic data analysis with triploid showed that the *PpnCKX1* transcript level had some dependence on the *PpnGRF5* (Fig. S2). In addition, tissue‐specific expression profiles of *PpnGRF5‐1/2* and *PpnCKX1* indicated that *PpnCKX1* transcript levels were inversely correlated with both *PpnGRF5‐1* and *PpnGRF‐2* (Fig. 2a–c). Based on these findings, we hypothesized that PpnGRF5‐1 directly suppresses *PpnCKX1*. To test this, we first analyzed the 3 kb proximal promoter region upstream and 100 bp region downstream of the translation start site of *CKX1* from the 84K poplar, and found that one *cis*‐element (TGTCAG) of GRF‐binding sites is present at two locations: one is 1710 bp upstream and the other is 60 bp downstream from the CKX1 start codon. We amplified *c.* 150 bp flanking regions of each motif (300 bp total) and named these *PagCKX1p‐1* (−1710 bp site) and *PagCKX1p‐2* (+60 bp site) (Fig. 5a). We then used regions from the promoter to drive a *LacZ* reporter gene in a Y1H system. We found that PpnGRF5‐1 stably interacted with the TGTCAG motif in the *PagCKX1* proximal promoter region. Mutations of this *cis*‐element sequence in the two *PagCKX1* promoter fragments abolished PpnGRF5‐1 binding, suggesting that this interaction is authentic (Fig. 5b). To further verify binding, an electrophoretic mobility shift assay (EMSA) was carried out to determine whether PpnGRF5‐1 could directly bind to the *PagCKX1* promoter *in vitro*. As shown in Fig. 5c, PpnGRF5‐1 specifically bound to the promoter fragments containing normal TGTCAG sequence, but not the promoter fragments with the mutated *cis*‐element sequence. These results verified the binding of PpnGRF5‐1 to the *PagCKX1* promoter.

**Fig. 6:**
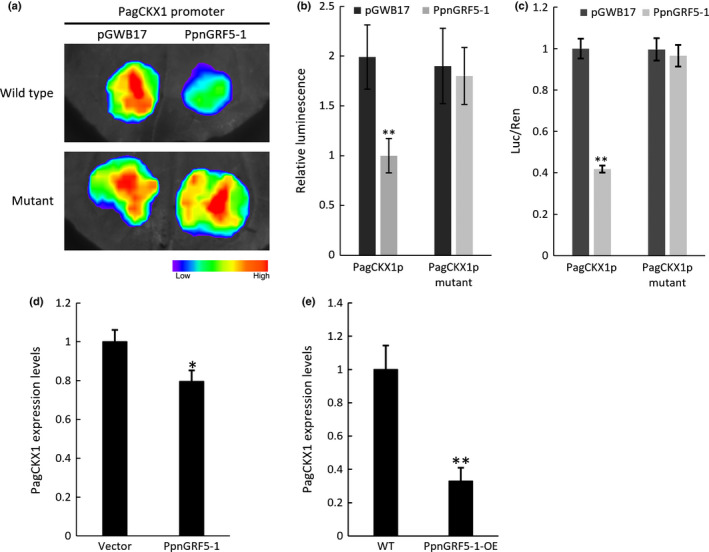
PpnGRF5‐1 directly represses the expression of the *PagCKX1* gene. (a) Transient expression assays show that PpnGRF5‐1 directly represses the expression of *PagCKX1*. Representative images of *Nicotiana benthamiana* leaves 48 h after infiltration were shown. (b) Quantitative analysis of luminescence intensity in (a). Values shown are mean ± SD (*n* = 5). **, *P* < 0.01 (by Student’s *t*‐test). Five independent determinations were assessed. (c) Dual‐LUC assay of *PagCKX1p::LUC* expression. The expression of REN was used as an internal control. LUC/REN ration represents the relative activity of the *PagCKX1* promoter. Values given are mean ± SD (*n* = 3). **, *P* < 0.01 (by Student’s *t*‐test). (d, e) RT‐qPCR analysis of *PagCKX1* expression. The RNA was extracted from 84K protoplasts transformed *PpnGRF5‐1* after 36 h, and from *PpnGRF5‐1‐*overexpression lines. Values shown are mean ± SD (*n* = 3). *, *P* < 0.05; **, *P* < 0.01 (determined by Student’s *t*‐test.)

Next, we examined whether *PpnGRF5‐1* could directly regulate the transcription of the target *PagCKX1* gene using a dual‐LUC reporter assay. GV3101, harboring the *35S::PpnGRF5‐1* effector and *PagCKX1p::LUC* reporter plasmids, was injected into *N. benthamiana* leaf epidermal cells. We found that the *PagCKX1p::LUC* reporter was activated in *N. benthamiana* leaves in the absence of *PpnGRF5‐1*, indicating that some endogenous factors in *N. benthamiana* activate the *PagCKX1p::LUC* reporter gene in mature tobacco leaves (Fig. 6a–c). Co‐expression of *PpnGRF5‐1* strongly inhibited the *LUC* reporter gene activities driven by the 3 kb poplar promoter of *PagCKX1*, but this inhibition was abolished by mutations in the TGTCAG motifs in this promoter (Fig. 6a–c). Next, we verified the inverse relationship between *PagCKX1* and *PpnGRF5‐1* expression in 84K mesophyll protoplasts and in poplar transgenic lines; *PpnGRF5‐1* was transiently overexpressed in the former and stably overexpressed in the latter. Thus, 36 h after transformation, a qRT‐PCR assay showed that the expression level of *PagCKX1* in 84K mesophyll protoplasts with *35S::PpnGRF5‐1‐GFP* was lower than those with *35S::GFP* (Fig. 6d). Consistent with this, *PagCKX1* transcription was suppressed in the fifth leaf of *PpnGRF5‐1‐OE* (Fig. 6e). We thus concluded that PpnGRF5‐1 binds to the *PagCKX1* promoter directly and represses its expression.

### Identification of DEGs in *PpnGRF5‐1‐OE* 84k poplar transgenic lines and GO enrichment analysis

The phenotypic changes, particularly the larger leaves in *PpnGRF5‐1‐OE* lines, prompted us to identify DEGs whose transcription was changed in apical buds. To do this, we ran RNA‐seq experiments with nine *PpnGRF5‐1‐OE* lines (*35S::PpnGRF5‐1‐eGFP‐nos*) and three WT 84K poplar replicates. The sequence reads yielded from these lines were aligned to the 84K poplar genome (Qiu *et al*., 2019), and the raw read counts of all genes were recovered. We compared three groups of transgenic lines (each with three lines) with low, medium, and high expression levels of *PpnGRF5‐1* to the three WT (Fig 4a) for DEG identification. We identified 2163 DEGs (corrected *P* < 0.05) by comparing the transgenic and WT 84K poplar lines (Supporting Information, Dataset S1). Of these DEGs, 1212 were upregulated while 951 were downregulated. The GO enrichment of the 2163 DEGs was performed using Pop’s pipeline and identified 280 biological processes; each was represented by a GO term. In the 2163 DEGs, the biological processes of leaf development (GO:0048366, 51 genes), leaf morphogenesis (GO:0009965, 23 genes), shoot system morphogenesis (GO:0010016, 42 genes), regulation of meristem development (GO:0048509, 23 genes), meristem growth (GO:0035266, 19 genes), regulation of meristem growth (GO:0010075, 18 genes), positive regulation of developmental process (GO:0051094, 10 genes), chlorophyll catabolic process (GO:0015996, 13 genes), chlorophyll metabolic process (GO:0015994, 20 genes), response to cytokinin (GO:0009735, 27 genes), hormone catabolic process (GO:0042447, four genes), and hormone transport (GO:0009914, 14 genes) were significantly enriched. Up to 21.8% DEGs can be functionally linked to morphogenesis, development, and growth of meristems and leaves, and nitrogen metabolism as well (Figs S7, S8).

### DAP‐seq identification of genes directly targeted by PpnGRF5‐1

We performed DAP‐seq to identify genes directly targeted by PpnGRF5‐1 by following the procedure described (Bartlett *et al*., 2017). The DAP‐seq analysis was performed using PpnGRF5‐1 protein and 84K genomic DNA with two biological replicates. Binding peaks of PpnGRF5‐1 were identified in each replicate separately and compared with those identified in control samples to eliminate nonspecific binding peaks. Peaks were considered for downstream analysis only if they were identified in both biological replicates. The analysis of resulting sequencing data resulted in 11 218 putative target sites bound by PpnGRF5‐1 protein *in vitro*. Obviously, the majority of the peaks identified (68.46%) were located in the proximal upstream regions including the 5′ untranslated region (5′UTR) and around the transcription start site (Fig. S9a,b). Of these 11 218 sites, 3031 putative target genes in the 84K poplar genome were shown to have promoter sequences enriched with the TGTCAG motif (Dataset S2; Fig. S10, *P*‐value = 0.05). We found that many binding sites (TGTCAG) of PpnGRF5‐1 in the 5′ proximal regions (including parts of 5′ coding regions) of some target genes are corresponding precisely to the peaks of read density (Fig. S11), which explicitly indicates that PpnGRF5‐1 bound to TGTCAG motif in DAP‐seq experiment.

### *PpnGRF5‐1*‐mediated ML‐hGRN in *PpnGRF5‐1* overexpression transgenic lines

**Fig. 7:**
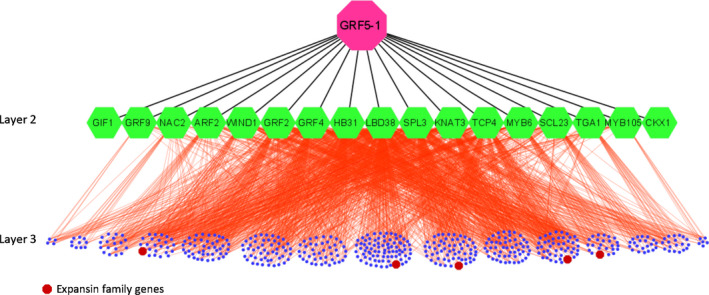
A three‐layered GRN mediated by PpnGRF5‐1. Each line represents a regulatory relationship inferred by top‐down GGM algorithm and validated by DAP‐seq, and each node in Layer 2 denotes a direct target gene while each dot at Layer 3 represents an indirect target gene of PpnGRF5‐1.

With 2163 DEGs identified from *PpnGRF5‐1‐OE* lines, we used our top‐down GGM algorithm (Lin *et al*., 2013; Wei, 2019) to infer a PpnGRF5‐1‐mediated ML‐hGRN. We first identified DEGs whose expression levels were tightly responsive to that of PpnGRF5‐1 using Fisher’s exact test (at low stringency, *P* < 0.15) and a probability method, as described earlier (Lin *et al*., 2013); 1397 PpnGRF5‐1‐responsive genes were chosen from the 2163 DEGs. PpnGRF5‐1 interfered with 858 of these 1397 PpnGRF5‐1‐responsive genes. Finally, we intersected these 858 genes with the PpnGRF5‐1 target genes identified via DAP‐seq and motif examination and identified 197 direct target genes of PpnGRF5‐1. Of these 197 genes, 27 were TFs (Dataset S3). The remaining 1966 DEGs were considered indirect targets of PpnGRF5‐1. Interestingly, *PagCKX1* and *PagGIF1* were also among these 197 genes. We selected 15 of the 27 TFs identified and repeated the two‐step procedure described earlier with the top‐down GGM algorithm to obtain target genes in the next layer using the expression profiles of these 15 TFs and 1966 indirect target genes. The PpnGRF5‐1‐mediated ML‐hGRN obtained is shown in Fig. 7.

In the ML‐hGRN, several crucial TFs, including *PagGRF2*, *PagGRF4*, *PagGIF1*, *PagARF2*, *PagMYB105*, *PagTCP4*, *PagLBD38*, *PagNAC2* and *PagSPL3*, involved in meristem initiation and maintenance, boundary specification, and leaf development were present in the second layer. The regulatory genes that were directly mediated by PpnGRF5‐1 indeed to a large degree support that PpnGRF5‐1 is a master regulator of leaf growth in triploid poplar (Dataset S3). For example, *PagTCP4*, which was downregulated almost two times in the *PpnGRF5‐1‐OE* lines and negatively regulated leaf development, had an inferred regulatory interaction with 15 leaf growth‐related genes, such as *PagTFPD*, *PagWIN1*, *PagHMGR1*, *PagPEL3*, *PagWRKY22*, *PagPLL4* and *PagERF9*. In the third layer, many functional genes, which were jointly regulated by the TFs in the second layer, were also enriched in cell division and expansion. For example, there was strong interference between the cell cycle‐regulating gene *PagCYCD3* and *PagARF2*, *PagGRF4*, and *PagMYB6*. The computational results presented here need to be validated experimentally.

## Discussion

Compared with diploids, almost all plant polyploids display much larger leaf sizes, which are determined by the coordinated regulation of cell division and cell growth/expansion (Gonzalez *et al*., 2012; Nelissen *et al*., 2016). Therefore, a study of cell division and expansion is essential to understand leaf growth regulatory mechanisms. In this study, we revealed that the larger leaves in triploid poplar resulted from both increased cell numbers and expanded cell sizes compared to those of full‐sib diploids. Overexpression of *PpnGRF5‐1* in 84k diploid poplar led to larger leaves that resembled those of triploids, indicating that *PpnGRF5‐1* and its downstream target genes are sufficient to drive leaf growth and produce triploid leaf phenotypes. In the PpnGRF5‐1‐mediated ML‐hGRN, 197 direct target genes including *PagCKX1*, *PagTCP4* and *PagGIF1* were identified (Fig. 7). We randomly selected 30 genes from these 197 and examined the presence of PpnGRF5‐1 binding motifs (TGTCAG) in their 5′ proximal regions (including parts of 5′ coding regions) where the peaks of read density yielded from DAP‐seq presented; 12 of them had the binding motif (Fig. S11). The reasons for lack of the binding motif in the proximal promoters of 18 other genes include: (1) the binding motifs of PpnGRF5‐1 have other variants; (2) binding of PpnGRF5‐1 on a specific motif sometime might be dependent on the upstream and downstream sequences around the motif or the presence of other co‐regulatory proteins; (3) DAP‐seq might have a fraction of false positives. In addition, the intersection of the DEGs from triploids and *PpnGRF5‐1‐OE* lines in comparison with the respective controls led to identification of 1245 common genes, which included *CKX1*, *TCP4*, *MAN7*, *CYP78A7*, *GIF1*, *HYR1* and *CRF11*. Indeed, these results suggest that *PpnGRF5‐1* regulate many of the same target genes in *PpnGRF5‐1*‐*OE* lines (diploids) and triploids where *PpnGRF5‐1* expression levels were elevated. The mechanism underlying the elevated expression of *PpnGRF5‐1* in triploids is unknown, but, one review (Chen & Ni, 2006) indicates the altered gene expression in polyploids occurs primarily through chromosomal remodeling and the RNA‐mediated gene regulation that ensues when subgenomes interact with one other.

**Fig. 8:**
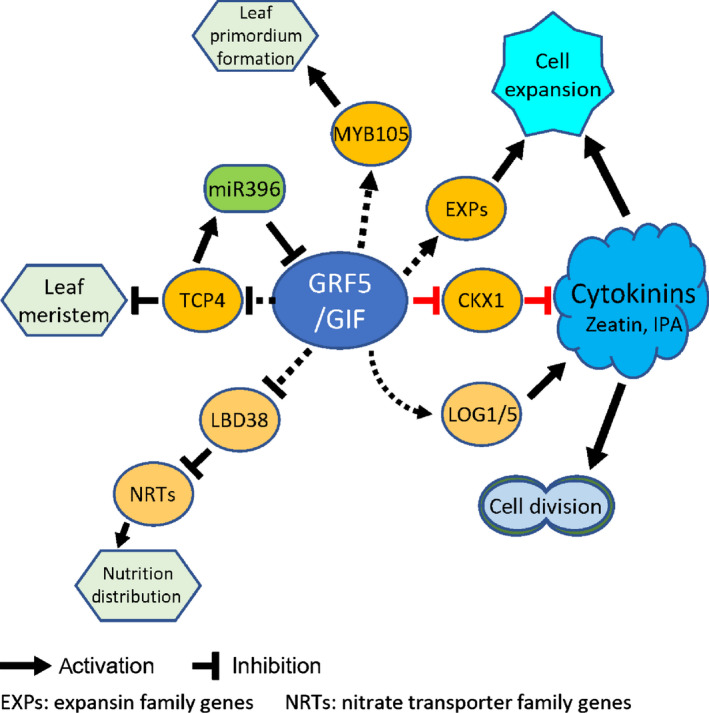
A holistic model that reflects the role of PpnGRF5‐1 in cell division and expansion. Each solid line denotes a direct regulatory relationship that has been proven by existing literature or our experiment data (Y1H and EMAS), whereas each dashed line denotes a potential regulatory relationship we predicted by combining top‐down GGM algorithm and DAP‐seq experiment.

Cytokinins generally function in promoting mitotic cell division (Schaller *et al*., 2014), regulating shoot apical meristem (SAM) activity, and organizing shoot architecture (Niemann *et al*., 2015). For leaf development, cytokinins are needed to maintain cell proliferation by blocking the transition to cell expansion and the onset of photosynthesis. In addition, cytokinins stimulate cell expansion and differentiation during the cell expansion phase (Skalak *et al*., 2019). Our study showed that cell numbers and cell sizes increased 14% and 43% in triploids compared to full‐sib diploids, respectively, and 19% and 82% in *PpnGRF5‐1‐OE* lines compared to WT, respectively. Currently, there is no evidence that *GRFs* directly regulate cytokinin synthesis genes, such as *IPTs* and *LOGs*, or cytokinin degradation genes such as *CKXs*. However, *AtGRFs* interact with *AtGIF1/AN3* to directly activate *AtCRF2* and repress *AtARR4* (Vercruyssen *et al*., 2014) in *Arabidopsis*, indicating that *AtGRFs* are involved in cytokinin signal transduction. In rice, overexpression of *OsGRF4* promotes six cell cycle genes and decreases two cytokinin dehydrogenase genes (*OsCKX5* and *OsCKX1*); but, the relationships between *OsGRF4* and the two *CKXs* were not characterized in the study where the elevated levels of cytokinins (iPR and *cis‐*zeatin) were observed (Sun *et al*., 2016). In this study, we ascertained that PpnGRF5‐1 directly regulated *PagCKX1*, leading to the increased zeatin, which is in agreement with an earlier report (Du *et al*., 2020), and IPA in the leaves of triploids (Fig 4g–i). Therefore, the escalation of cytokinins by augmenting *PpnGRF5‐1* expression is one of the major mechanisms underpinning the leaf growth advantages in triploid poplar. In *Arabidopsis thaliana*, the cytokinin signal transduction pathway involves hybrid histidine protein kinases (e.g. *AHK2*, *AHK3*, and *AHK4*) that function as cytokinin receptors (Higuchi *et al*., 2004; Lomin *et al*., 2015), histidine phosphotransfer proteins (*AHPs*) (Suzuki *et al*., 1998), and nuclear response regulators (*ARRs*) (To *et al*., 2004; Mason *et al*., 2005) as well as cytokinin activating genes, *LOG1/5*. In this study, we found that the counterparts of *PagAHK3/4*, *PagAHP5*, *PagARR24* and *PagLOG1/5* were present in the DEGs in *PpnGRF5‐1‐OE* lines (Fig. 8; Dataset S1). These results indicate that the cytokinin signaling pathway was augmented to promote leaf growth and expansion as the cytokinins were accumulated in apical buds and young leaves owing to *PpnGRF5‐1* overexpression.

Among 197 direct target genes of PpnGRF5‐1, some function in cell expansion, for example, *ARF2* (Schruff *et al*., 2006), *TCP4* (Schommer *et al*., 2014), *SPL3* (Usami *et al*., 2009), *SCL23* (Yoon *et al*., 2016), *WIND1* (Iwase *et al*., 2011), *GRF2* and *GRF4* (Liebsch & Palatnik, 2020). They work together to form multiple chains of regulatory command (or regulatory cascades) to promote cell expansion. One function of *expansin* (*EXP*) genes is to promote cell expansion (Marowa *et al*., 2016). Several *EXP* genes (*EXP2*, *EXLA2*, *EXP3*, *EXPL3* and *EXP11*) in *PpnGRF5‐1* overexpression lines were significantly upregulated (log_2_ fold change is: 1.1, 1.6, 1.0, 1.2 and 1.9, respectively) (Fig. 8; Dataset S1). These *EXP* genes are present at the low terminal in PpnGRF5‐1 mediated GRN and are indirect target genes of PpnGRF5‐1(Fig. 7).

Multiple target genes of PpnGRF5‐1, which include *PagGRF2*, *PagGRF4*, and *PagGRF9*, *PagGIF1*, *PagTCP4*, *PagMYB105*, and *PagLBD38*, function in developmental, metabolic, and growth processes. The GRF‐GIF duo associated with the SWI2/SNF2 complex regulates transcription of target genes, including a mutual auto‐activation between GRFs and GIFs through a positive feedback loop in *Arabidopsis* *thaliana*, rice, and maize (Kim & Tsukaya, 2015; Zhang *et al*., 2018; Kim, 2019). For example, a GIF gene mutant, *Atan3*, downregulates *AtGRF6* and *AtGRF9*, but overexpression of *AtAN3* significantly upregulates *AtGRF3*, *AtGRF5*, and *AtGRF6* (Vercruyssen *et al*., 2014). We showed that PpnGRF5‐1 interacted with PpnGIF1, PpnGIF2, and PpnGIF3, which could increase the regulatory diversity and capacity of PpnGRF5‐1. Some results obtained from *PpnGRF5‐1*‐*OE* lines implicate that there were some complications. For example, a few *PagGRFs* (*PagGRF2*, *PagGRF4*, and *PagGRF9*) were activated to some degree (log_2_ fold change is: 1.1, 0.7 and 2.4, respectively), whereas two *GRFs*, *PagGRF1* and *PagGRF7*, and two *GIFs*, *PagGIF1* and *PagGIF3*, decreased (log_2_ fold changes were −1.5 and −1.7, −1.0 and −1.1, respectively) in the *PpnGRF5‐1‐OE* lines (Dataset S1). Therefore, a further understanding of the regulation of leaf growth and development of PpnGRF5‐1 requires to investigate if there exist some regulatory circuits among PpnGRF5‐1 and other GRFs and also among PpnGRF5‐1 and GIFs.

Recent studies showed that *AtTCP4* gradually restricts the activity of leaf meristem in *Arabidopsis thaliana* to marginal and basal domains (Alvarez *et al*., 2016) and causes leaf cells within the transition zone to commit to exiting proliferation and entering differentiation (Challa *et al*., 2019). In addition, *AtTCP4* induces *miR396* that represses the activity of *AtGRFs* (Rodriguez *et al*., 2010). In our study, PpnGRF5‐1 bound to the promoter of *PagTCP4* and downregulated it in *PpnGRF5‐1‐OE* lines (log_2_ fold change is −0.5). Furthermore, our computational analysis with psRNATarget (Dai *et al*., 2018) showed that *PpnGRF5‐1* is targeted by miRNA396 in poplar with high affinity (Expection: 3.0, UPE: 17.036). We thus hypothesize that *PpnGRF5‐1* delays cell differentiation and increases cell division in the leaf marginal and basal domains by directly suppressing the expression of *PpnTCP4,* indicating the presence of a negative feedback loop between *GRFs* and *TCPs* (Fig. 8).

Recently, *OsGRF4* and DELLA protein *SLR1* were reported to counteract each other to regulate NH_4_^+^ uptake and assimilation (Li *et al*., 2018); OsGRF4 promotes both nitrogen uptake and assimilation, whereas *SLR1* inhibits these processes. In our study, we found that *PpnGRF5‐1* regulated *PagLBD38* (Dataset S3), whose counterpart in *Arabidopsis thaliana* (*AtLBD38*) represses many other known N‐responsive genes including key genes, for example, nitrate transporters (NRTs), required for nitrate uptake and assimilation (Rubin *et al*., 2009). In *PpnGRF5‐1*‐overexpression lines, the transcript abundance of *PagLBD38* was lower than that of WT (log_2_ fold change is −1.1), and genes involved in nitrate transport, including *PagNRT1*.*5* and *PagNRT1*.*7*, were upregulated (log_2_ fold change is 3.2‐ and 1.5, respectively) in apical buds. These results give an inkling that *PpnGRF5‐1* can regulate nutritional supplements through *PagLBD38* during leaf development, and further research is needed to verify this (Fig. 8).

Another upregulated target gene of PpnGRF5‐1 is *PagMYB105* (log_2_ fold change is 0.8) (Datasets S1 and S3). Its counterpart in *Arabidopsis thaliana* (*AtMYB105*) was classified into the 21st subfamily of the myeloblastosis oncoprotein (MYB) family. *AtMYB105*/*AtLOF2* and *AtMYB117/AtLOF1* (subgroup 21) function redundantly to control lateral organ separation and axillary meristem formation (Lee *et al*., 2009). *AtLOFs* control cell division and expansion at the boundary between cauline leaf and axillary branch and regulate organ separation and axillary meristem formation (Lee *et al*., 2009). Based on the evidence, further research is needed to investigate if PpnGRF5‐1 controls leaf primordium formation and separation from ground meristems through regulating *PagMYB105* (Fig. 8).

### Conclusion

In conclusion, we identified a high hierarchical TF, *PpnGRF5‐1*, which governs the meristematic potential of proliferative and formative cells during leaf development in poplar. In triploid poplar, *PpnGRF5‐1* expression was specifically enhanced leading to significantly larger leaf blades. Transformation of *PpnGRF5‐1* into diploid 84K poplar followed by network construction and analysis revealed that *PpnGRF5‐1* controlled leaf growth and development by mediating an ML‐hGRN, in which multiple top‐down chains‐of‐command enable *PpnGRF5‐1* to directly regulate a battery of hub‐regulators and indirectly regulate a large number of growth and developmental genes. We specifically tested one of these, the *PpnGRF5‐1*‐*PpnCKX1* regulatory command chain, which modulated the concentrations of two active cytokinins, zeatin and IPA, in young leaves. In addition, our analyses revealed other potential *PpnGRF5‐1* regulatory chains of command through which various growth and developmental pathways and processes were coordinately regulated to determine final leaf sizes through enhancing cell division and expansion.

## Author contributions

WW performed most experiments, and XK generated the triploid poplars and performed RNA‐seq on triploids. HW identified *GRF5* from triploids and guided the overall research. JL and WZ helped with the DAP‐seq experiment and data analysis; QW and QL helped with the protein–protein interaction experiment. KL and KD helped with gene transformation; WW and HW wrote the manuscript.

## Supporting information


**Dataset S1** List of differentially expressed genes identified in 3‐month‐old *PpnGRF5‐1* overexpression transgenic plants.
**Dataset S2** List of PpnGRF5‐1 DNA affinity purification sequencing (DAP‐seq).

**Dataset S3** List of PpnGRF5‐1 direct target genes.

**Fig. S1** Leaf area and cell area of fifth, sixth, and seventh leaves from 5‐month‐old triploid and diploid poplars.
**Fig. S2** *PpnGRF5* and *PpnCKX1* expression levels and correlation in two transcriptomic data sets from triploid (generated by first division restitution (FDR) gametes) and full‐sib diploid poplars.
**Fig. S3** Phylogenetic analysis of *growth‐regulating factor* (*GRF*) genes in *Populus trichocarpa* and *Arabidopsis thaliana*.
**Fig. S4** PpnGRF5‐1 protein domains and potential to act as a transcription factor.
**Fig. S5** Subcellular localization of PpnGRF5‐1‐GFP induced fluorescence in the 84K poplar leaf mesophyll protoplasts.
**Fig. S6** The various phenotypes and the expression levels of *PpnGRF5‐1* in its overexpression (OE) transgenic lines.
**Fig. S7** Enriched gene ontology (GO) in the differentially expressed genes identified from the apical buds of the 3‐month‐old *PpnGRF5‐1* overexpression lines as compared with the 84K wild‐type (WT).
**Fig. S8** The top 30 biological processes resulting from gene ontology (GO) enrichment analysis on the differentially expressed genes (DEGs) identified from *PpnGRF5‐1*‐overexpression transgenic lines (apical buds) in comparison with the 84K wild type (WT).
**Fig. S9** The distribution of PpnGRF5‐1 DNA affinity purification sequencing (DAP‐seq) reads in different genic and intergenic regions.
**Fig. S10** Identification of overrepresented variants of PpnGRF5‐1 binding motifs from PpnGRF5‐1 DNA affinity purification sequencing (DAP‐seq) data using Homer software (v.4.11).
**Fig. S11** Mapping the genome‐wide binding sites of PpnGRF5‐1 in the 84K poplar genome using DNA affinity purification sequencing (DAP‐seq).
**Methods S1** PpnGRF5‐1 using top‐down graphic Gaussian model (top‐down GGM) algorithm.
**Methods S2** RNA isolation, RT‐PCR and qRT‐PCR.
**Methods S3** Transcriptional activation analysis in yeast cells.
**Methods S4** Yeast one‐hybrid assays.
**Methods S5** Electrophoretic mobility shift assay (EMSA).
**Methods S6** DNA affinity purification sequencing (DAP‐seq) and data analysis.
**Methods S7** Dual‐luciferase assay.
**Methods S8** Yeast two‐hybrid assays.
**Methods S9** GST (glutathione‐S‐transferase)‐fusion protein pull‐down assay and western blotting.
**Methods S10** Split luciferase complementation assay.
**Table S1** All primer sequences used in this study.
**Table S2** The height, diameter and the fifth leaf area of 5‐month‐old *PpnGRF5‐1* overexpression transgenic lines.
Please note: Wiley Blackwell are not responsible for the content or functionality of any Supporting Information supplied by the authors. Any queries (other than missing material) should be directed to the *New Phytologist* Central Office.

## Data Availability

The one transcriptomic data set was submitted to Genome Sequence Archive with an accession no. CRA003633. DAP‐seq and RNA‐seq data of *PpnGRF5‐1* transgenic overexpression lines reported in this manuscript have been submitted to the NCBI SRA database under the accession no. SRP265938.
