# Supplementary material for: Growth‐regulating factor 5 (GRF5)‐mediated gene regulatory network promotes leaf growth and expansion in poplar
Source: New Phytol. 2021 Feb 14;230(2):612–28. doi: 10.1111/nph.17179 (PMC8048564; doi:10.1111/nph.17179)
Supplement: Supplementary file 3 — Fig. S1 Leaf area and cell area of fifth, sixth, and seventh leaves from 5‐month‐old triploid and diploid poplars. Fig. S2 PpnGRF5 and PpnCKX1 expression levels and correlation in two transcriptomic data sets from triploid (generated by first division restitution (FDR) gametes) and full‐sib diploid poplars. Fig. S3 Phylogenetic analysis of growth‐regulating factor (GRF) genes in Populus trichocarpa and Arabidopsis thaliana. Fig. S4 PpnGRF5‐1 protein domains and potential to act as a transcription factor. Fig. S5 Subcellular localization of PpnGRF5‐1‐GFP induced fluorescence in the 84K poplar leaf mesophyll protoplasts. Fig. S6 The various phenotypes and the expression levels of PpnGRF5‐1 in its overexpression (OE) transgenic lines. Fig. S7 Enriched gene ontology (GO) in the differentially expressed genes identified from the apical buds of the 3‐month‐old PpnGRF5‐1 overexpression lines as compared with the 84K wild‐type (WT). Fig. S8 The top 30 biological processes resulting from gene ontology (GO) enrichment analysis on the differentially expressed genes (DEGs) identified from PpnGRF5‐1‐overexpression transgenic lines (apical buds) in comparison with the 84K wild type (WT). Fig. S9 The distribution of PpnGRF5‐1 DNA affinity purification sequencing (DAP‐seq) reads in different genic and intergenic regions. Fig. S10 Identification of overrepresented variants of PpnGRF5‐1 binding motifs from PpnGRF5‐1 DNA affinity purification sequencing (DAP‐seq) data using Homer software (v.4.11). Fig. S11 Mapping the genome‐wide binding sites of PpnGRF5‐1 in the 84K poplar genome using DNA affinity purification sequencing (DAP‐seq). Methods S1 PpnGRF5‐1 using top‐down graphic Gaussian model (top‐down GGM) algorithm. Methods S2 RNA isolation, RT‐PCR and qRT‐PCR. Methods S3 Transcriptional activation analysis in yeast cells. Methods S4 Yeast one‐hybrid assays. Methods S5 Electrophoretic mobility shift assay (EMSA). Methods S6 DNA affinity purification sequencing (DAP‐seq) and data ana [file NPH-230-612-s003.pdf]

New Phytologist **Supporting Information**

Article title: **Growth-Regulating Factor 5 (GRF5) mediated gene regulatory network promotes leaf growth and expansion in poplar**

Authors: **Wenqi Wu[1], Jiang Li[1], Qiao Wang[2], Kaiwen Lv[3], Kang Du[1], Wenli Zhang[4], Quanzi Li[2], Xiangyang Kang[1ξ], Hairong Wei[5ξ]**

Article acceptance date: 28 December 2020

The following Supporting Information is available for this article:

Fig. S1 Leaf area and cell area of 5th, 6th, and 7th leaves from five-month-old triploid and diploid poplars.

Fig. S2 *PpnGRF5* and *PpnCKX1* expression levels and correlation in two transcriptomic data sets from triploid (generated by first division restitution gametes (FDR)) and full-sib diploid poplars.

Fig. S3 Phylogenetic analysis of *growth-regulating factor* (*GRF*) genes in *Populus trichocarpa* and *Arabidopsis thaliana*.

Fig. S4 PpnGRF5-1 protein domains and potential to act as a transcription factor.

Fig. S5 Subcellular localization of PpnGRF5-1-GFP induced fluorescence in the 84K poplar leaf mesophyll protoplasts.

Fig. S6 The various phenotypes and the expression levels of *PpnGRF5-1* in its overexpression (OE) transgenic lines.

Fig. S7 Enriched gene ontologies (GO) in the differentially expressed genes identified from the apical buds of the three-month-old PpnGRF5-1 overexpression lines as compared with the 84K wild type (WT).

Fig. S8 The top 30 biological processes resulting from gene ontology (GO) enrichment analysis on the differentially expressed genes (DEGs) identified from *PpnGRF5-1*-overexpression lines (apical buds) in comparison with the 84K wild type (WT).

Fig. S9 The distribution of PpnGRF5-1 DNA affinity purification sequencing (DAP-seq) reads in different genic and intergenic regions.

Fig. S10 Identification of overrepresented variants of PpnGRF5-1 binding motifs from PpnGRF5-1

DNA affinity purification sequencing (DAP-seq) data using Homer software (v4.11).

Fig. S11 Mapping the genome-wide binding sites of PpnGRF5-1 in the 84K poplar genome using DNA affinity purification sequencing (DAP-seq).

Table S1 All primer sequences used in this study.

Table S2 The height, diameter and the fifth leaf area of five-month-old *PpnGRF5-1* overexpression transgenic lines.

Methods S1 PpnGRF5-1 using top-down Gaussian graphical model (top-down GGM) algorithm

Methods S2 RNA isolation, RT-PCR, and qRT-PCR

Methods S3 Transcriptional activation analysis in yeast cells

Methods S4 Yeast one-hybrid assays

Methods S5 Electrophoretic Mobility Shift Assay (EMSA)

Methods S6 DNA affinity purification sequencing (DAP-seq) and data analysis

Methods S7 Dual-luciferase assay

Methods S8 Yeast two-hybrid assays

Methods S9 GST (glutathione-S-transferase)-fusion protein pull-down assay and western blotting

Methods S10 Split luciferase complementation assay

Fig. S1 Leaf area and cell area of 5th, 6th, and 7th leaves from five-month-old triploid and diploid poplars. Leaf area and cell area of fifth leaves from three-month-old diploid and triploid poplars (30 leaves were measured).Values represent the mean ± SD.

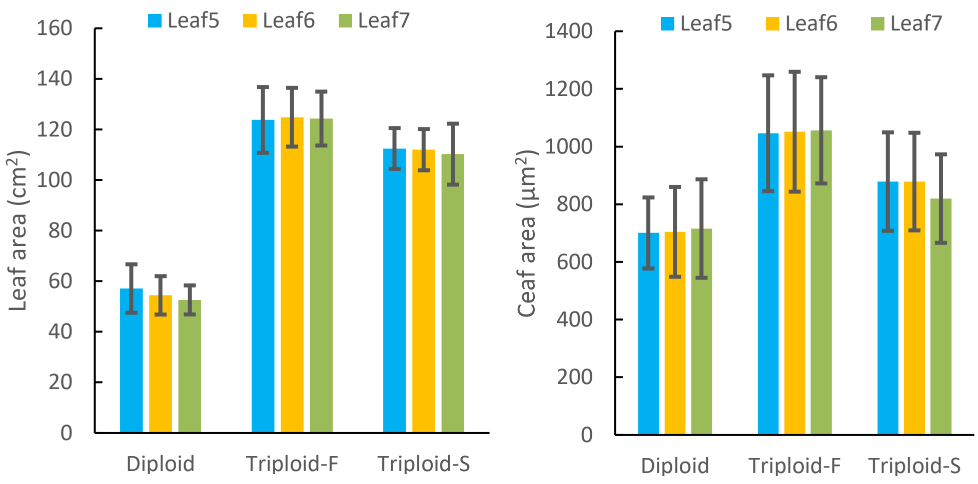

Fig. S2 *PpnGRF5* and *PpnCKX1* expression levels and correlation in two transcriptomic data sets from triploid (generated by first division restitution gametes (FDR)) and full-sib diploid poplars. The data were normalized with Trimmed Mean of M-values (TMM) method from the edgeR package. P-value* is the corrected p-value (false discovery rate method) of differentially expressed gene identified by voom algorithm; NS, not significant.

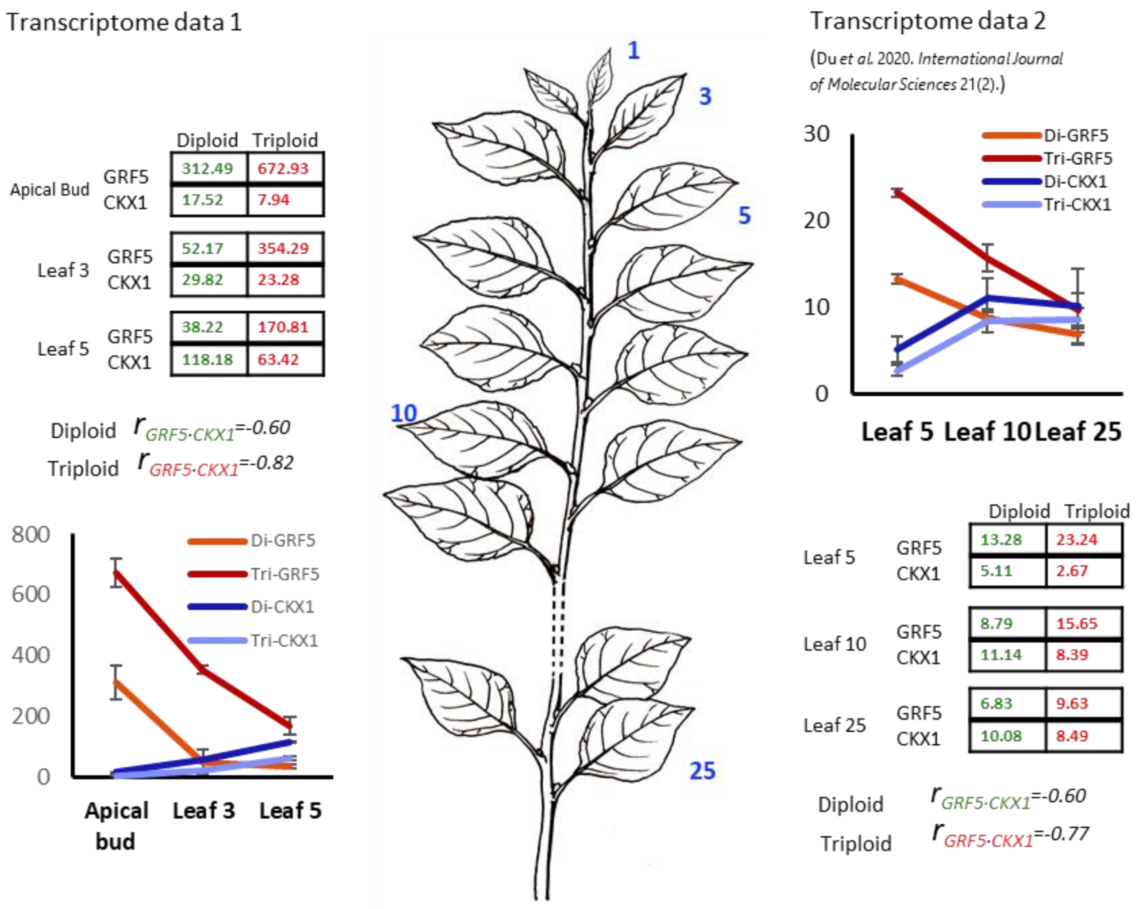

Fig. S3 Phylogenetic analysis of *growth-regulating factor* (*GRF*) genes in *Populus trichocarpa* and *Arabidopsis thaliana*.

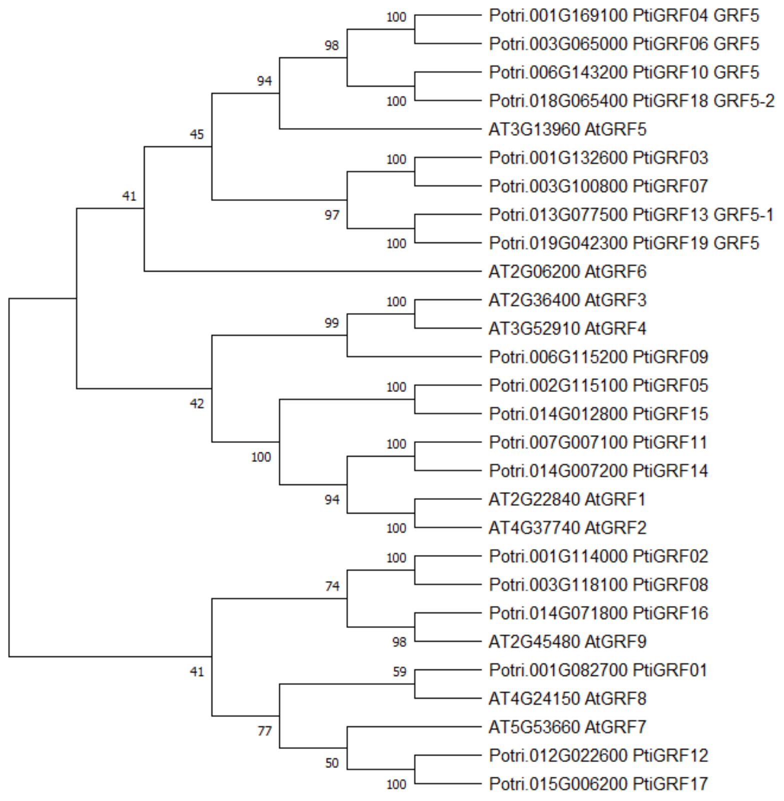

Fig. S4 PpnGRF5-1 protein domains and potential to act as a transcription factor. (a) Structure of PpnGRF5-1 protein contained one QLQ and one WRC domain, and the less conserved TQL motif was located within the C-terminal region of *PpnGRF5-1*. (b) Transactivation activity assays of PpnGRF5-1. Different DNA fragments of PpnGRF5-1 were fused to the sequence encoding GAL4-DBD and introduced into AH109 yeast cells. The vector pGBKT7 was used as a negative control. Yeast were spotted onto nutritional selective medium (SD/-Trp, SD/-Trp-His, or SD/-Trp-Ade-His) and allowed to grow at 30℃ for three days.

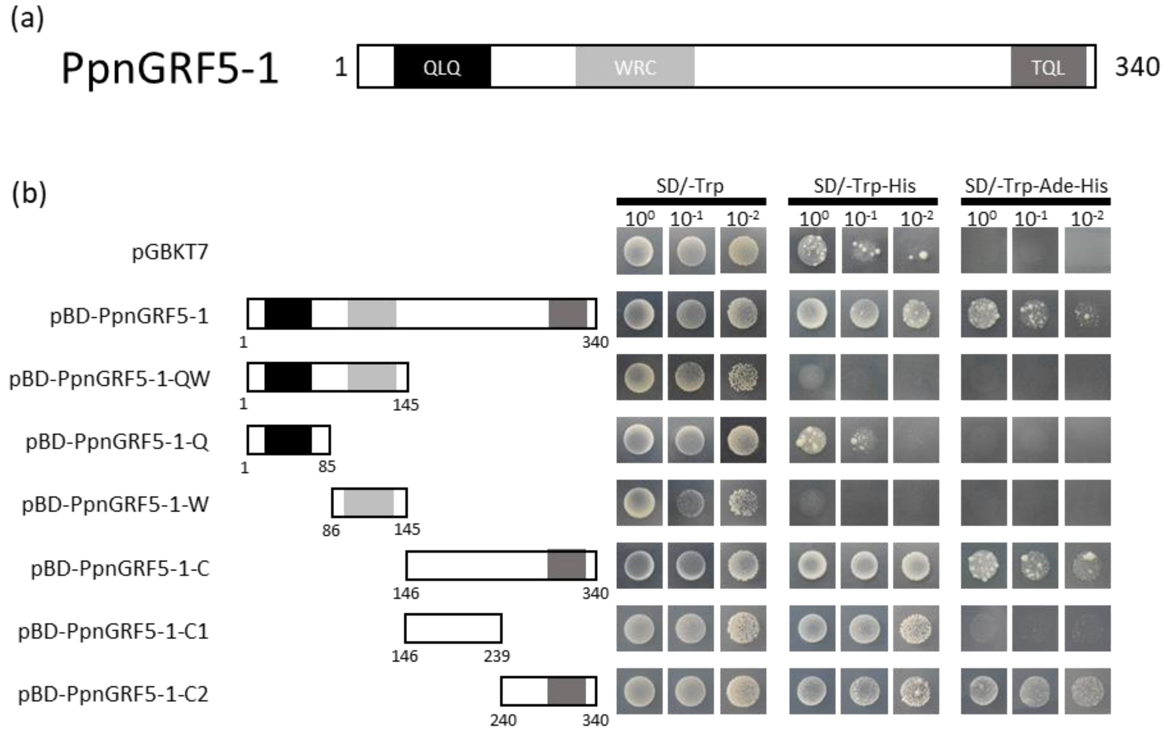

Fig. S5 Subcellular localization of PpnGRF5-1-GFP induced fluorescence in the 84K poplar leaf mesophyll protoplasts.

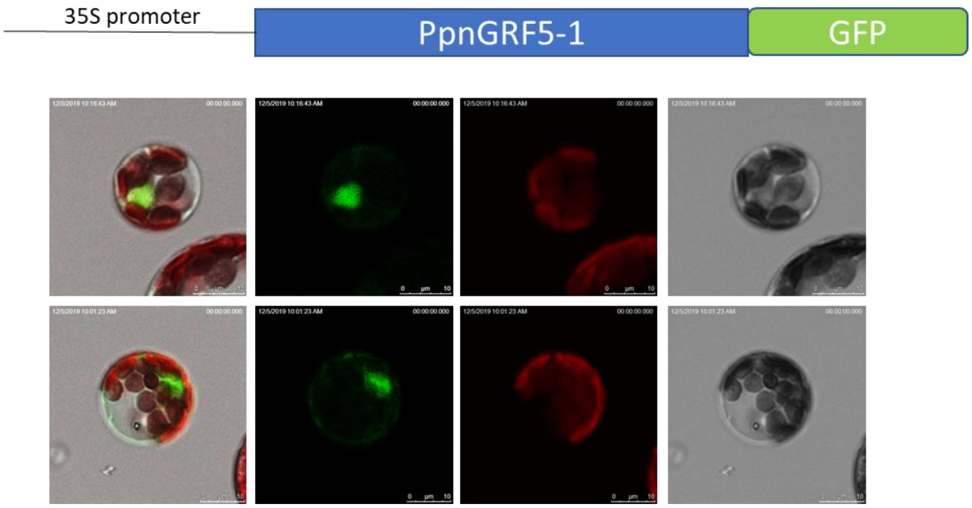

Fig. S6 The various phenotypes and the expression levels of *PpnGRF5-1* in its overexpression (OE) transgenic lines. (a) The fifth leaves of three-month-old *PpnGRF5-1* overexpression transgenic lines and 84K wild type (WT) poplar trees from tissue couture grown in soil in pots. Scale bar 2 cm. (b) Bar chart: the relative expression levels of *PpnGRF5-1* in the apical buds (including a couple of unexpended small leaves within the buds), the third and the fifth leaves of *PpnGRF5-1* overexpression transgenic lines. Different letters denote statistically significant differences resulting from Tukey’s range test following two-way ANOVA. Line plot: leaf areas of the fifth leaves of three-month-old *PpnGRF5-1* overexpression transgenic lines and 84K WT poplar trees from tissue couture grown in soil in pots. Values represent the mean ± SD.

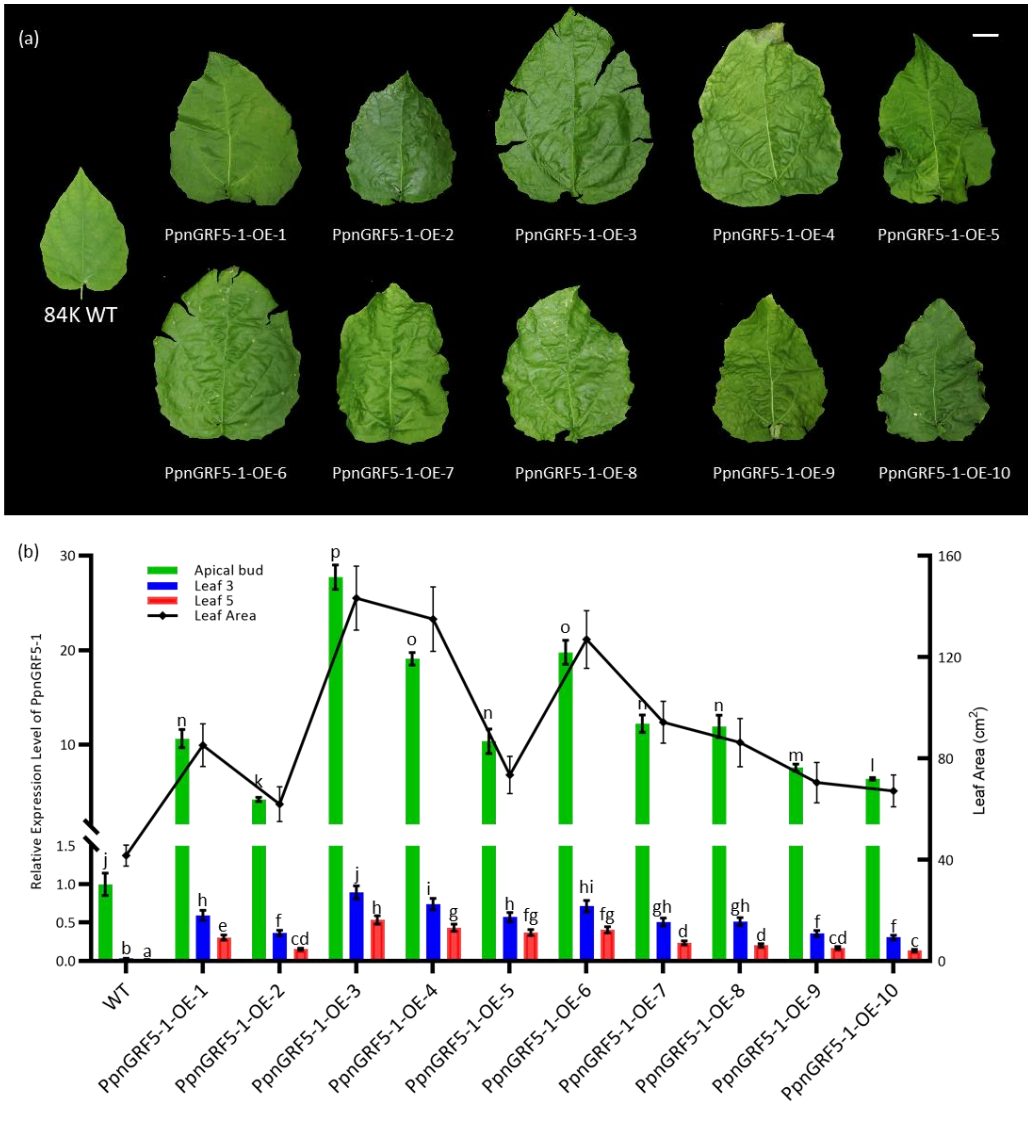

Fig. S7 Enriched gene ontologies (GO) in the differentially expressed genes identified from the apical buds of the three-month-old *PpnGRF5-1* overexpression lines as compared with the 84K wild type (WT). Each node represents an enriched GO term and a bigger node size represents a larger number of DEGs. Each node color denotes a certain scale of p-value of a GO term.

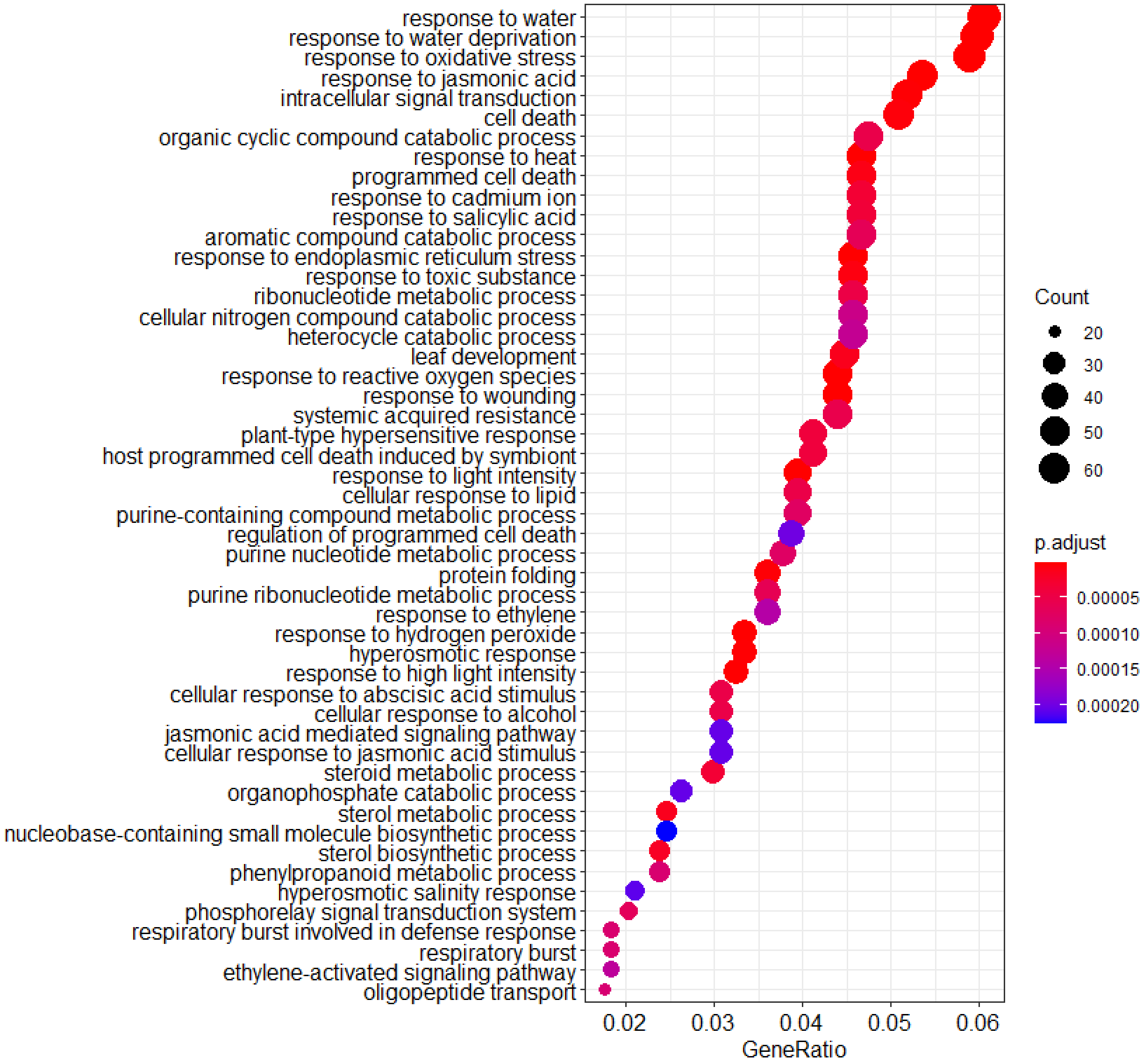

Fig. S8 The top 30 biological processes resulting from gene ontology (GO) enrichment analysis on the differentially expressed genes (DEGs) identified from *PpnGRF5-1*-overexpression lines (apical buds) in comparison with the 84k wild type (WT). Each node represents an enriched GO term and a bigger node size represents a larger number of DEGs. Each node color denotes a certain scale of p-value of a GO term. Each line represents overlapping DEGs between the two GO terms; a bigger edge width represents a bigger number of overlapping DEGs. The enrichment was analysed using R module named clusterProfiler (v 3.14.3).

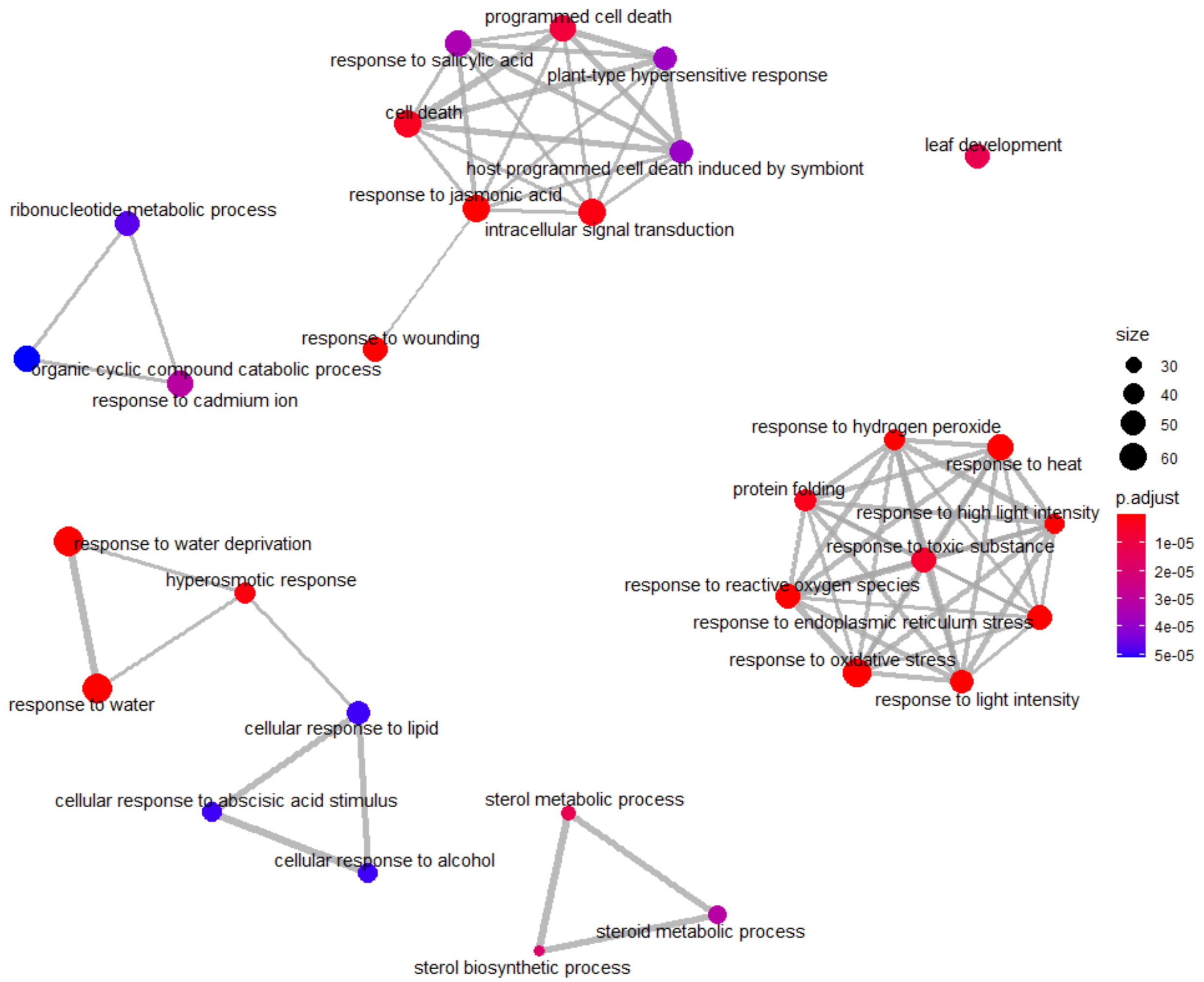

Fig. S9 The distribution of PpnGRF5-1 DNA affinity purification sequencing (DAP-seq) reads in different genic and intergenic regions. (a) The pie chart represents the percentages of DAP-seq reads within different genic and intergenic regions. (b) Frequency of DAP-seq read counts at transcription start sites (TSS) and flanking regions. Values represent the mean ± SD.

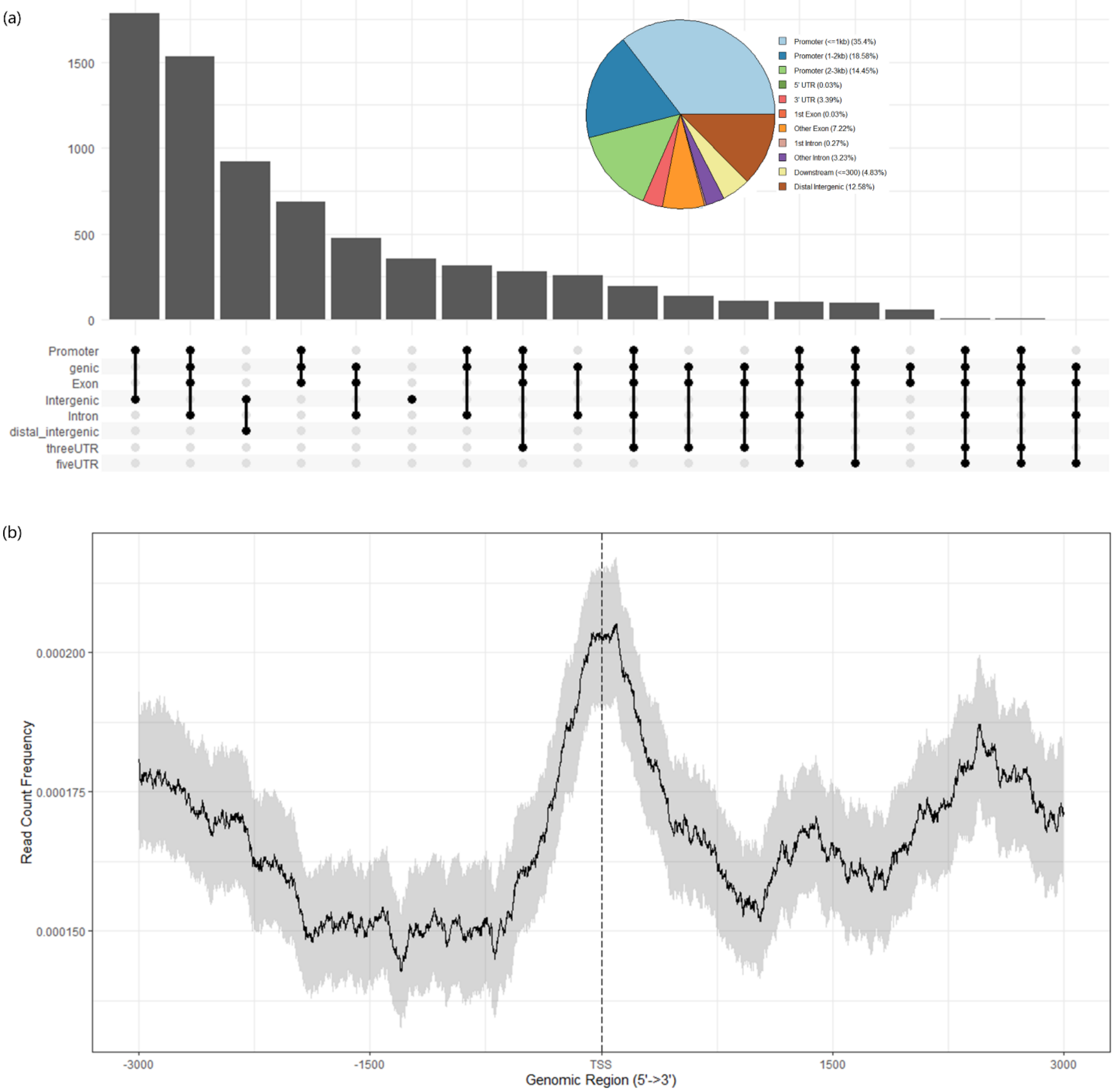

Fig. S10 Identification of overrepresented variants of PpnGRF5-1 binding motifs from PpnGRF5-1 DNA affinity purification sequencing (DAP-seq) data using Homer software (v4.11).

Rank	Motif	P-value	log P-pvalue	% of Targets	% of Background	STD(Bg STD)
1	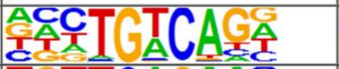	1e-6314	-1.454e+04	52.93%	13.14%	33.1bp (73.3bp)
2	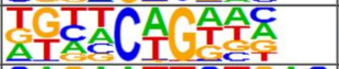	1e-486	-1.119e+03	70.75%	58.06%	53.9bp (71.2bp)
3	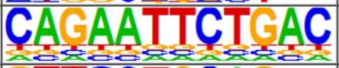	1e-442	-1.019e+03	26.25%	16.35%	51.5bp (70.6bp)
4	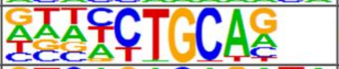	1e-279	-6.431e+02	40.33%	30.93%	51.4bp (73.8bp)
5	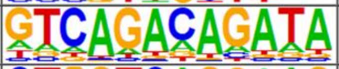	1e-210	-4.845e+02	5.46%	2.39%	51.7bp (72.0bp)
6	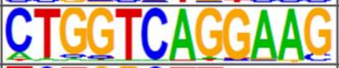	1e-70	-1.632e+02	0.17%	0.00%	46.1bp (0.0bp)
7	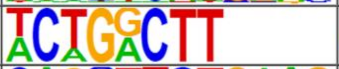	1e-60	-1.404e+02	5.84%	3.93%	53.9bp (70.0bp)
8	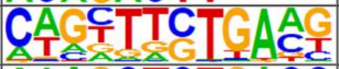	1e-57	-1.334e+02	0.25%	0.02%	44.2bp (13.3bp)
9	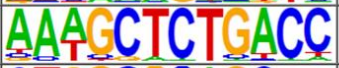	1e-56	-1.307e+02	0.23%	0.02%	49.8bp (24.9bp)
10	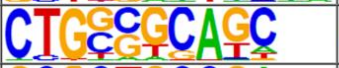	1e-52	-1.205e+02	2.08%	1.09%	50.4bp (64.4bp)
11	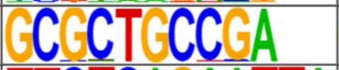	1e-46	-1.061e+02	0.12%	0.01%	56.0bp (22.6bp)
12	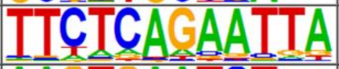	1e-35	-8.211e+01	0.45%	0.13%	55.3bp (62.1bp)
13	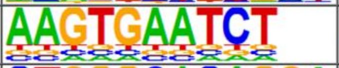	1e-28	-6.450e+01	0.08%	0.01%	56.4bp (20.6bp)
14	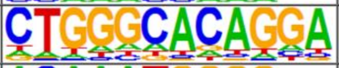	1e-27	-6.305e+01	0.15%	0.02%	53.1bp (48.4bp)
15	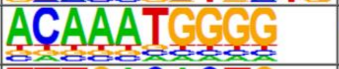	1e-21	-4.860e+01	0.07%	0.01%	55.5bp (19.7bp)
16	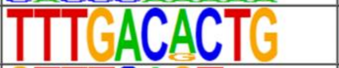	1e-19	-4.393e+01	0.17%	0.04%	58.4bp (56.8bp)
17	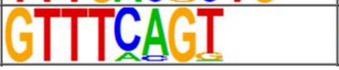	1e-17	-3.976e+01	3.33%	2.54%	56.2bp (67.4bp)

Fig. S11 Mapping the genome-wide binding sites of PpnGRF5-1 in the 84K poplar genome using DNA affinity purification sequencing (DAP-seq). Upper panel: The peaks around the binding sites (TGTCAG) of PpnGRF5-1 in the upstream proximal promoters and coding regions of PpnGRF5-1's direct target genes. Each red triangle represents a PpnGRF5-1 binding site. Lower panel: the exact locations of PpnGRF5-1 binding sites in the above-mentioned target genes.

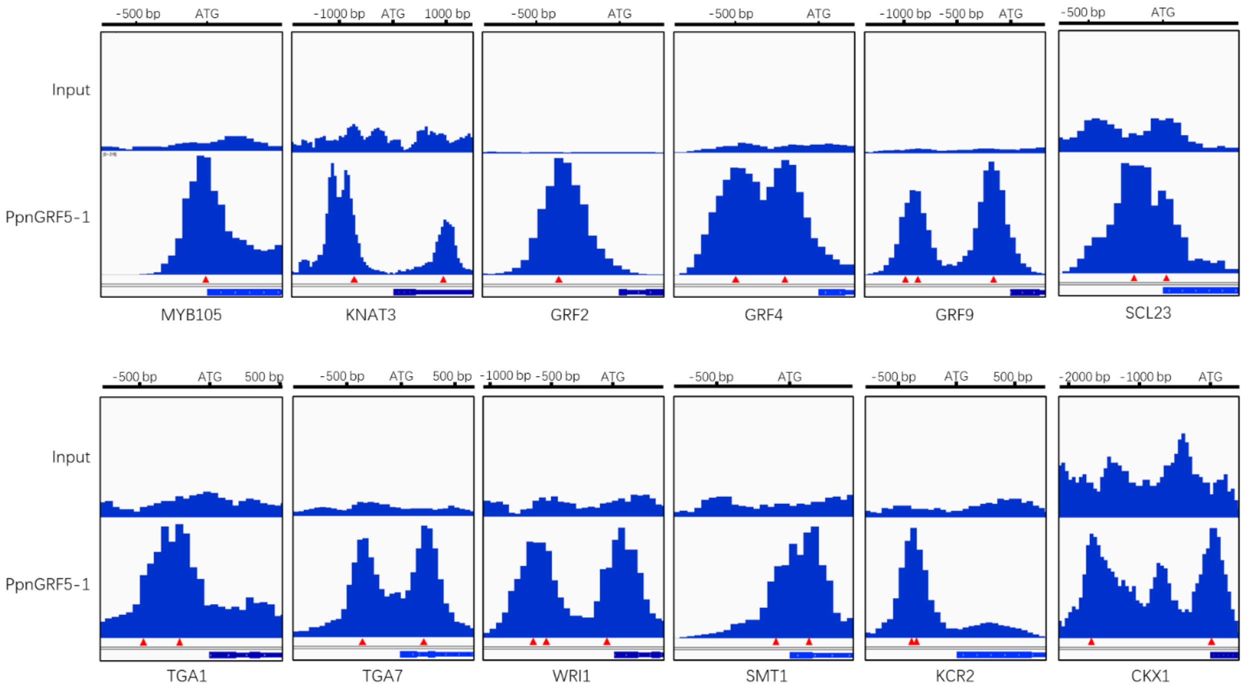

Gene	Chr	motif 1 location	motif 2 location	motif 3 location
MYB105	08	-22		
KNAT3	18	988	-857	
GRF2	03	-315		
GRF4	06	-541	-210	
GRF9	14	-201	-858	-1011
SCL23	06	-230	20	
TGA1	05	-187	-432	
TGA7	02	-373	202	
WRI1	10	-58	-552	-620
SMT1	01	-87	148	
KCR2	10	-377	-404	
CKX1	06	-1729	64	

Table S1 All primer sequences used in this study

Primer ID	Primer Sequence (5’->3’)	Purpose
GRF5-1-Q-F	CAAGAAAGCCTGTGGAACTTAC	qPCR of GRF5-1
GRF5-1-Q-R	GGGTTAGAGAGGTTTCTGTTGA	qPCR of GRF5-1
GRF5-2-Q-F	TCAGAGGAGCTTTGAATCCATT	qPCR of GRF5-2
GRF5-2-Q-R	ATCCACCTTCTTGCCATAGAAA	qPCR of GRF5-2
CKX1-Q-F	CAATCTTCTGTCAACCCAAGTG	qPCR of CKX1-Q
CKX1-Q-R	CTGTTAGCTTTGAAGTGGAACC	qPCR of CKX1-Q
ACTIN-Q-F	AAACTGTAATGGTCCTCCCTCCG	qPCR of ACTIN-Q
ACTIN-Q-R	GCATCATCACAATCACTCTCCGA	qPCR of ACTIN-Q
PpnGRF5-1-1F	ATGAATAGTGGTGGTGCAGG	Cloning GRF5-1 coding region
PpnGRF5-1-1023R	CTAGTTATCTCGTGGAGAACGACAAC	Cloning GRF5-1 coding region
pBD-PpnGRF5-1-1F	GAATTCCCGGGGATCATGAATAGTGGT GGTGCAGGAG	Cloning GRF5-1 coding region into pGBKT7 vector
pBD-PpnGRF5-1-1023R	GCAGGTCGACGGATCTCAGTTATCTCGT GGAGAACG	Cloning GRF5-1 coding region into pGBKT7 vector
pBD-PpnGRF5-1-261F	GAATTCCCGGGGATCCTATTGCACTTTC TATGGCA	Cloning GRF5-1 coding region into pGBKT7 vector
pBD-PpnGRF5-1-280R	GCAGGTCGACGGATCTCATGCCATAGA AAGTGCAATAG	Cloning GRF5-1 coding region into pGBKT7 vector
pBD-PpnGRF5-1-421F	GAATTCCCGGGGATCTCACAAACCATG ACACAGTCA	Cloning GRF5-1 coding region into pGBKT7 vector
pBD-PpnGRF5-1-441R	GCAGGTCGACGGATCTCATGACTGTGT CATGGTTTGTGA	Cloning GRF5-1 coding region into pGBKT7 vector
pBD-PpnGRF5-1-721F	GAATTCCCGGGGATCTTGATGCAATCC AGAGTCTC	Cloning GRF5-1 coding region into pGBKT7 vector
pBD-PpnGRF5-1-742R	GCAGGTCGACGGATCATGAGACTCTGG ATTGCATC	Cloning GRF5-1 coding region into pGBKT7 vector
pUC35S-GRF1-1F	CACGGGGGACTCTAGATGAATAGTGGT GGTGCAGGAG	Cloning GRF5-1 coding region into pUC-35S::GFP vector
pUC35S-GRF1-1020R	CCATGTCGACCTCGACGTTATCTCGTGG AGAACG	Cloning GRF5-1 coding region into pUC-35S::GFP vector
pAD-PpnGRF5-1-1F	CATCGATACGGGATCATGAATAGTGGT GGTGCAGGAG	Cloning GRF5-1 coding region into pGADT7 vector
pAD-PpnGRF5-1-1023R	CGAGCTCGATGGATCTCAGTTATCTCGT GGAGAACG	Cloning GRF5-1 coding region into pGADT7 vector
pBD-PpnGIF1-1F	GAATTCCCGGGGATCATGCAACAGCAC CTGATGCAG	Cloning GIF1 coding region into pGBKT7 vector
pBD-PpnGIF1-669R	GCAGGTCGACGGATCTCAATTCCCATC ATCTGCAG	Cloning GIF1 coding region into pGBKT7 vector
pBD-PpnGIF2-1F	GAATTCCCGGGGATCATGCAGCAGCCA CCGCAAATG	Cloning GIF2 coding region into pGBKT7 vector
pBD-PpnGIF2-627R	GCAGGTCGACGGATCTCATTTTGCATCC TCGGAAC	Cloning GIF2 coding region into pGBKT7 vector
pBD-PpnGIF3-1F	GAATTCCCGGGGATCATGCAACAGCAC CTGATGCAG	Cloning GIF3 coding region into pGBKT7 vector
pBD-PpnGIF3-663R	GCAGGTCGACGGATCTCAGTTCCCATCT TCAGCAG	Cloning GIF3 coding region into pGBKT7 vector
pYN-PpnGRF5-1-1F	CGCCACTAGTGGATCCATGAATAGTGG TGGTGCAGGAG	Cloning GRF5-1 coding region into pSPYNE(R) vector

Primer ID	Primer Sequence (5’->3’)	Purpose
pYN-PpnGRF5-1-1020R	TCCATCCCGGGAGCGGTGTTATCTCGTG GAGAACG	Cloning GRF5-1 coding region into pSPYNE(R)vector
pYC-PpnGIF1-1F	CGCCACTAGTGGATCCATGCAACAGCA CCTGATGCAG	Cloning GIF1 coding region into pSPYCE(M) vector
pYC-PpnGIF1-666R	GAGCGGTACCCTCGAGATTCCCATCATC TGCAGA	Cloning GIF1 coding region into pSPYCE(M) vector
pYC-PpnGIF2-1F	CGCCACTAGTGGATCCATGCAGCAGCC ACCGCAAATG	Cloning GIF2 coding region into pSPYCE(M) vector
pYC-PpnGIF2-624R	GAGCGGTACCCTCGAGTTTTGCATCCTC GGAACC	Cloning GIF2 coding region into pSPYCE(M) vector
pYC-PpnGIF3-1F	CGCCACTAGTGGATCCATGCAACAGCA CCTGATGCAG	Cloning GIF3 coding region into pSPYCE(M) vector
pYC-PpnGIF3-660R	GAGCGGTACCCTCGAGGTTCCCATCTTC AGCAGA	Cloning GIF3 coding region into pSPYCE(M) vector
pGEX-PpnGRF5-1-1F	GGTGGTGGTGGAATTATGAATAGTGGT GGTGCAGGAG	Cloning GRF5-1 coding region into pGEX-KG vector
pGEX-PpnGRF5-1-1023R	CACGATGAATAAGCTTCAGTTATCTCGT GGAGAACG	Cloning GRF5-1 coding region into pGEX-KG vector
pET-PpnGIF1-1F	TGGCTGATATCGGATCCATGCAACAGC ACCTGATGCAG	Cloning GIF1 coding region into pET28a vector
pET-PpnGIF1-666R	AGTGCGGCCGCAAGCTTGATTCCCATC ATCTGCAGA	Cloning GIF1 coding region into pET28a vector
pET-PpnGIF2-1F	TGGCTGATATCGGATCCATGCAGCAGC CACCGCAAATG	Cloning GIF2 coding region into pET28a vector
pET-PpnGIF2-624R	AGTGCGGCCGCAAGCTTGTTTTGCATCC TCGGAACC	Cloning GIF2 coding region into pET28a vector
pET-PpnGIF3-1F	TGGCTGATATCGGATCCATGCAACAGC ACCTGATGCAG	Cloning GIF3 coding region into pET28a vector
pET-PpnGIF3-660R	AGTGCGGCCGCAAGCTTGGTTCCCATCT TCAGCAGA	Cloning GIF3 coding region into pET28a vector
pBI-PpnGRF5-1-1F	CACGGGGGACTCTAGATGAATAGTGGT GGTGCAGGAG	Cloning GRF5-1 coding region into pBI121-GFP vector
pBI-PpnGRF5-1-1020R	ATACTAGTCAGTCGACCGTTATCTCGTG GAGAACG	Cloning GRF5-1 coding region into pBI121-GFP vector
pLac-PpnCKX1(-)2458F	TCGGAATTCGAGCTCGATTGCTTCCAAA CCGCTG	Cloning CKX1 promoter into pLacZi2μ vector
pLac-PpnCKX1-213R	GTCGACAGATCCCCGGATGTAGAACTG CCGATGGG	Cloning CKX1 promoter into pLacZi2μ vector
pLac-PpnCKX1(-)1860F	TCGGAATTCGAGCTCGGTTTGTTGGGCA CAAAAAGAG	Cloning CKX1 promoter into pLacZi2μ vector
pLac-PpnCKX1(-)1560R	GTCGACAGATCCCCGGTTAAGGTCAGA CACCATTCAAG	Cloning CKX1 promoter into pLacZi2μ vector
pLac-PpnCKX1(-)91F	TCGGAATTCGAGCTCGTCTCCTTCCTTC CCTTTCTCGC	Cloning CKX1 promoter into pLacZi2μ vector
PpnCKX1-M(-)1731F	TTCCCGCATTCAAACGCTACTCCTCAAG TTCACAGTTTC	Mutagenesis of the first motif of CKX1 promoter
PpnCKX1-M(-)1695R	ACTGTGAACTTGAGGAGTAGCGTTTGA ATGCGGGAAACA	Mutagenesis of the first motif of CKX1 promoter
PpnCKX1-M-44F	TTCCGACCAATCTTCGCTACTCCCAAGT GTGATTCCATA	Mutagenesis of the second motif of CKX1 promoter
PpnCKX1-M-80R	GGAATCACACTTGGGAGTAGCGAAGAT TGGTCGGAACAA	Mutagenesis of the second motif of CKX1 promoter

Primer ID	Primer Sequence (5’->3’)	Purpose
pGreen-PpnCKX1(-)2458F	CGGTATCGATAAGCTATTGCTTCCAAAC CGCTGCG	Cloning CKX1 promoter into pGreenII 0800 LUC vector
pGreen-PpnCKX1-213R	TTGGCGTCTTCCATGGGATGTAGAACTG CCGATGGGA	Cloning CKX1 promoter into pGreenII 0800 LUC vector
EMSA-CKX1-1-F	ACCTTTTTGGTGTTTCCCGCATTCAAAC TGTCAGCCTCAAGTTCACAGTTTCTCCC CTTT	Probe for the first motif of CKX1 EMSA
EMSA-CKX1-1-R	AAAGGGGAGAAACTGTGAACTTGAGGC TGACAGTTTGAATGCGGGAAACACCAA AAAGGT	Probe for the first motif of CKX1 EMSA
EMSA-CKX1-1m-F	ACCTTTTTGGTGTTTCCCGCATTCAAAC GCTACTCCTCAAGTTCACAGTTTCTCCC CTTT	Mutant probe for the first motif of CKX1 EMSA
EMSA-CKX1-1m-R	AAAGGGGAGAAACTGTGAACTTGAGGA GTAGCGTTTGAATGCGGGAAACACCAA AAAGGT	Mutant probe for the first motif of CKX1 EMSA
EMSA-CKX1-2-F	AATCAACCTTTGTTCCGACCAATCTTCT GTCAGCCCAAGTGTGATTCCATATAAG AGCTC	Probe for the second motif of CKX1 EMSA
EMSA-CKX1-2-R	GAGCTCTTATATGGAATCACACTTGGGC TGACAGAAGATTGGTCGGAACAAAGGT TGATT	Probe for the second motif of CKX1 EMSA
EMSA-CKX1-2m-F	AATCAACCTTTGTTCCGACCAATCTTCG CTACTCCCAAGTGTGATTCCATATAAGA GCTC	Mutant probe for the second motif of CKX1 EMSA
EMSA-CKX1-2m-R	GAGCTCTTATATGGAATCACACTTGGG AGTAGCGAAGATTGGTCGGAACAAAGG TTGATT	Mutant probe for the second motif of CKX1 EMSA
pGWB17-PpnGRF5-1-1F	CACGGGGGACTCTAGATGAATAGTGGT GGTGCAGGAG	Cloning GRF5-1 coding region into pGWB17 vector
pGWB17-PpnGRF5-1-1020R	TTTTGTTCACCGTTAGTTATCTCGTGGA GAACG	Cloning GRF5-1 coding region into pGWB17 vector

Table S2 The height, diameter and the fifth leaf area of five-month-old *PpnGRF5-1* overexpression transgenic lines. Different letters denote statistically significant differences resulting from Tukey's range test following one-way ANOVA. Values represent the mean ± SD.

	Height (cm)	Diameter (mm)	Leaf Area (cm^2)
WT	93.56±5.52 a	4.04±0.26 a	45.72±3.94 a
PpnGRF5-1-OE-1	94.96±4.59 a	4.72±0.41 b	90.03±5.58 c
PpnGRF5-1-OE-2	94.13±7.29 a	4.59±0.3 ab	63.81±5.00 b
PpnGRF5-1-OE-3	99.28±4.83 a	4.71±0.17 b	130.90±7.22 d
PpnGRF5-1-OE-4	91.29±4.08 a	4.70±0.31 b	124.30±7.45 d
PpnGRF5-1-OE-5	91.91±4.76 a	4.74±0.32 b	82.34±4.39 c
PpnGRF5-1-OE-6	94.35±5.60 a	4.80±0.19 b	117.10±8.12 d
PpnGRF5-1-OE-7	94.55±3.83 a	4.72±0.43 b	85.47±5.07 c
PpnGRF5-1-OE-8	96.87±4.95 a	4.72±0.17 b	90.44±7.23 c
PpnGRF5-1-OE-9	94.20±4.62 a	4.59±0.19 ab	73.19±2.63 bc
PpnGRF5-1-OE-10	100.70±3.58 a	4.48±0.30 ab	67.75±5.49 b

Methods S1 Top-down Gaussian graphical model (top-down GGM) algorithm

The two-step procedure for construction of a TF-mediated ML-hGRN was adopted. The step-by-step procedure was thoroughly described in two of our earlier publications (Lin *et al.*, 2013; Wei, 2019). In the first step, we identified the TF-responsive genes, the ones whose expression profiles were highly concordant with TF's. This was done by integration of Fisher's exact test and a probability-based method. In the second step, the interference frequency between TF and each TF-responsive candidate target gene was determined by a top-down GGM algorithm (Lin *et al.*, 2013; Wei, 2019). Briefly, given a combination of TF (z) and a pair of TF-responsive genes (x and y), the significance of interference of z on x and y was determined by testing if the presence of *z* makes the correlation between *x* and *y* become more or less significant. This was accomplished by examining if d = r_{xy} - $r_{xy|z}$ was significant by the multivariate delta method (MacKinnon *et al.*, 2002), where $r_{xy|z}$ is the Spearman's partial correlation coefficient of x and y given z while r_{xy} is Spearman's correlation rho of x and y. If the p-value of *d* was significant, we then concluded that *z* interfered with both x and y, which were recorded as *z* interfered with x and *z* interfered with y. After all combinations of different *x* and *y* were tested with *z*, the interference frequency of *z* with each responsive gene was determined by counting the significant interference in all combinations. PpnGRF5-1-responsive candidate target genes that were interfered by z with the highest frequencies were retained, and subsequently intersected with PpnGRF5-1 target genes derived from DAP-seq experiment, and only those that were in the interaction and also possessed PpnGRF5-1 binding motifs in their proximal promoters were kept at the second layer of GRN as the direct target of PpnGRF5-1. The next layer (namely, the third layer) is extended in a top-down fashion only from the TFs present in the second layer (direct targets of PpnGRF5-1) by recursively calling the top-down GGM algorithm. More detailed procedure of top-down GGM algorithm can be found from two of our early publications (Lin *et al.*, 2013; Wei, 2019), and also from two recent application studies (Chen *et al.*, 2019; Wei *et al.*, 2020).

Methods S2 RNA isolation, RT-PCR, and RT-qPCR

Total RNA was isolated from the collected materials with TRIzol reagent (Thermo Fisher, USA, cat 15596026), and then treated with RNase-free DNase I (Thermo Fisher, USA, cat EN0521) according to the manufacturer's instructions. Full-length cDNA was then reverse transcribed using a cDNA synthesis kit (Tiangen, China, cat KR106). qPCR was performed according to the manufacturer's instructions (TRANSGEN, China, cat AQ142-21) in a total volume of 25 μL on the Applied Biosystems 7500 real-time PCR system according to the manufacturer's manual. All real-time PCR reactions were repeated at least three times. The level of *Actin* gene (*Potri.001G309500*) transcript was used as an internal control. The specific primers of these genes were obtained by directly querying the prime data qPrimerDB (https://biodb.swu.edu.cn/qprimerdb) (Lu *et al.*, 2018). Three technical replicates and three biological

replicates were performed on all reactions. The ΔΔCt algorithm was used for calculating relative gene expression. Primers are listed in Table S1.

Methods S3 Transcriptional activation analysis in yeast cells

For transcriptional activation activity assays, the different domain of *PpnGRF5-1* was fused with GAL4 DNA-binding domain in the pGBKT7 vector (Clontech, USA, cat 630443) and transformed into the yeast strain AH109 with Ade2 and His reporter genes, respectively. The empty pGBKT7 vector was used as a negative control. Transformed strains were confirmed by PCR and then plated on selective synthetic dropout (SD) media without Trp (SD/-Trp), without Trp and His (SD/-Trp-His), or without Trp, His, and Ade (SD/-Trp-His-Ade) with 5 mM 3-AT, to determine their survivability. All transcriptional activation assays were performed in three replications. Primers are listed in Table S1.

Methods S4 Yeast one-hybrid assays

The full-length sequence of *PpnGRF5-1* was amplified and fused into the activation domain (AD) in the pJG4-5 vector (Clontech) to generate *pJG4-5-PpnGRF5-1* construct. Fragments containing the two putative PpnGRF5-1 binding sites of 'TGTCAG' or all of the substitution mutants of the two binding sites in the CKX1 promoter were independently amplified and fused into the vector pLacZi2μ (Lin *et al.*, 2007). Briefly, the yeast strain EGY48 cells were transformed with PpnGRF5-1 fusion constructs and various LacZ reporter plasmids. Then the yeasts were plated on synthetic dropout medium containing X-gal but without tryptophan and uracil for blue color development. Primers are listed in Table S1. All assays were performed in three replications.

Methods S5 Electrophoretic Mobility Shift Assay (EMSA)

EMSA was performed to examine if PpnGRF5-1 can bind to the promoter of *CKX1*. To do this, two complementary 60-bp long oligonucleotides containing the binding *cis*-elements were separately synthesized and then labeled with biotin using EMSA Probe Biotin Labeling Kit (Beyotime, China, cat GS008). GST and GST-PpnGRF5-1 recombinant proteins were expressed in the *Escherichia coli* BL21 (DE3) strain and then purified using GST-tag Protein Purification Kit (Beyotime, China, cat P2262). DNA gel mobility shift assay was performed using the EMSA kit (Beyotime, China, cat GS009) following the manufacturer's protocol. Briefly, the DNA probes and proteins were co-incubated in the reaction buffer at room temperature. We added a specific competitor (non-biotin) and non-specific competitor (mutated) probes into the reaction mixture for competition reaction. After incubation, the reaction mixture was separated by 6% native polyacrylamide gel, and then, labeled DNA was detected using the Biostep Celvin S420 system (Biostep, German). All assays were performed in three replications. Primers are listed in Table S1.

Methods S6 DNA affinity purification sequencing (DAP-seq) and data analysis

DAP-seq was performed as described previously (Bartlett *et al.*, 2017). The coding sequence of PpnGRF5-1 was cloned into the vector pFN19K. Then PpnGRF5-1 protein expression was expressed using TnT® Coupled Wheat Germ Extract System (Promega, USA, L4130). Genomic DNA (gDNA) was extracted from apical bus (meristem + tiny unopened leaves) that harvested from three-month-old 84K populus following the procedure of the DNeasy plant mini kit (Qiagen, Germany, cat 69104). The gDNA was sonicated to a fragment size of 200 to 800 bp. Halo-PpnGRF5-1 protein was bound to anti-Halo monoclonal antibody agarose beads (Promega, USA, cat G9211) and incubated with 200 ng of fragmented gDNA for 1 hour at room temperature. After incubation, the beads were washed and DNA was recovered. Samples were pooled and sequenced on an Illumina Novaseq 6000 platform and an average of 150 bp paired-end reads were generated from each library. A total of 10–30 million reads were obtained for each sample. Two biological replicates were analyzed. Primers are listed in Table S1.

The sequencing reads were trimmed using Trimmomatic with the following parameters: ILLUMINACLIP:TruSeq3-PE.fa:2:30:10:8:true LEADING:20 TRAILING:20 SLIDINGWINDOW:4:20 MINLEN:50. Trimmed reads were mapped to the 84k poplar genome using Bowtie2 software (v2.3.5). Mapped reads were filtered to obtain uniquely mapping reads using SAMtools software. Uniquely mapping reads were used for all subsequent analyses. Peak calling was done with MACS2 software. Association of DAP-seq peaks located within 2 kb upstream or downstream of the transcription start site (TSS) were analyzed using BEDtools according to the General Feature Format (GFF) files. Visualization of peaks coverage over chromosomes and profiles of peaks binding to TSS regions were analyzed using ChIPseeker software (v1.22.1). Motif discovery was performed using Homer software (v4.11).

Methods S7 Dual-luciferase assay

Firefly luciferase (LUC), under the control of the promoter of *CKX1*, was inserted into the reporter vector pGreenII 0800-LUC vector (Henriksson *et al.*, 2005). To generate the effector vector, the full-length *PpnGRF5-1* coding sequence was cloned into the vector pGWB17, to obtain *35S::PpnGRF5-1-nos* construct. Both the effector and reporter constructs were transformed into GV3101. *N. benthamiana* plants were transfected with both the effector and reporter, as previously described (Wang *et al.*, 2011). Luciferase activity was detected as in 'split luciferase complementation assay'. The luciferase activity quantification was also measured using the dual-luciferase reporter assay system (Promega, USA, cat E1910), and Renilla luciferase was used in normalization. Primers are listed in Table S1. All assays were performed in three replications.

Methods S8 Yeast two-hybrid assays

To examine if PpnGRF5-1 protein can interact with PpnGIFs proteins, the PpnGRF5-1 and PpnGIFs sequences were independently amplified and cloned into vectors pGBKT7 and pGADT7. The plasmids were co-transformed into the yeast strain AH109 mediated by PEG4000. The yeast two-hybrid was performed according to Matchmaker Gold Yeast Two-Hybrid System (Clontech, USA). All assays were performed in three replications. Primers are listed in Table S1.

Methods S9 GST (glutathione-S-transferase)-fusion protein pull-down assay and western blotting

The interaction of PpnGRF5-1 and PpnGIFs was determined by the GST-pulldown assay. The GST, GST-PpnGRF5-1, and PpnGIFs-His_6 recombinant proteins, were expressed in the *Escherichia coli* BL21 (DE3) strain and then purified using GST-tag Protein Purification Kit (Beyotime, China, cat P2262) and His-tag Protein Purification Kit (Beyotime, China, cat P2226), respectively. The equal amounts of GST and GST-PpnGRF5-1 proteins that had been coupled to glutathione-sepharose beads were incubated with recombinant PpnGIFs-His_6 with continuous rotation at 4 °C for 1 hour. Washing with phosphate-buffered saline (PBS) was followed to remove non-specific bound proteins for 5 times. After that, the proteins bound to the beads were eluted in sodium dodecyl sulfate (SDS) sample buffer with boiling. The sample mixes were separated in sodium dodecyl sulfate polyacrylamide gel electrophoresis (SDS-PAGE) and then transferred onto polyvinylidene fluoride (PVDF) membrane (Merck Millipore) by using electro-blotter. The membranes were blocked with 5% (w/ v) skim milk through incubation at 37 °C for 2 hours. The blots were incubated with primary antibodies overnight at 4°C in blocking buffer (5% BSA (bovine serum albumin) add to TBST buffer). Following that, alkaline phosphatase-conjugated secondary antibody that targeted for the specific primary antibody was added. Target bands were visualized by the reaction with nitro blue tetrazolium (NBT) and 5-bromo-4-chloro-3-indolyl phosphate (BCIP). Several antibodies were used are anti-GST (cat ab19256), anti-His (cat ab137839), and IgG H&L (cat ab6722), which were all purchased from Abcam (Cambridge, UK). All assays were performed in three replications. Primers are listed in Table S1.

Methods S10 Split luciferase complementation assay

The split-luciferase complementation assay was used to test if *PpnGRF5-1* and *PpnGIFs* interact with each other. The coding sequence of all *PpnGRF5-1* and *PpnGIFs* without the stop codon was inserted into the vector (pCAMBIA-NLuc and pCAMBIA-CLuc) to generate the *35S::PpnGRF5-1-nLUC-nos*, *35S::cLUC-PpnGIF1- nos, 35S::cLUC-PpnGIF2-nos* and *35S::cLUC-PpnGIF3-nos* fused constructs. These constructs were transformed into GV3101. *Nicotiana benthamiana* leaves were co-injected with mixed bacterial cultures using a needleless syringe. The leaves were collected after 3 days under long-day white-light conditions and infiltrated with 150 μg/ml luciferin solution. Luciferase activity was detected with a 30 s exposure time, 4 × 4 binning, slow readout, and high gain using Night SHADE LB 985 system (Berthold,

Germany). The bioluminescence intensities were calculated using Indigo software (v2.0.3.0, Berthold Technologies). Five independent replicates were used for the data analysis. All assays were performed in three replications. Primers are listed in Table S1.

References

Bartlett A, O'Malley RC, Huang S-SC, Galli M, Nery JR, Gallavotti A, Ecker JR. 2017. Mapping genome-wide transcription-factor binding sites using DAP-seq. *Nature Protocols* **12**(8): 1659-1672.

Chen H, Wang JP, Liu H, Li H, Lin Y-CJ, Shi R, Yang C, Gao J, Zhou C, Li Q, et al. 2019. Hierarchical Transcription Factor and Chromatin Binding Network for Wood Formation in Black Cottonwood (Populus trichocarpa). *Plant Cell* **31**(3): 602-626.

Du K, Liao T, Ren Y, Geng X, Kang X. 2020. Molecular Mechanism of Vegetative Growth Advantage in Allotriploid *Populus*. *International Journal of Molecular Sciences* **21**(2): 441.

Henriksson E, Olsson ASB, Johannesson H, Johansson H, Hanson J, Engstrom P, Soderman E. 2005. Homeodomain leucine zipper class I genes in Arabidopsis. Expression patterns and phylogenetic relationships. *Plant Physiology* **139**(1): 509-518.

Lin RC, Ding L, Casola C, Ripoll DR, Feschotte C, Wang HY. 2007. Transposase-derived transcription factors regulate light signaling in *Arabidopsis*. *Science* **318**(5854): 1302-1305.

Lin YC, Li W, Sun Y-H, Kumari S, Wei HR, Li QZ, Tunlaya-Anukit S, Sederoff RR, Chiang VL. 2013. SND1 transcription factor-directed quantitative functional hierarchical genetic regulatory network in wood formation in *Populus trichocarpa*. *Plant Cell* **25**(11): 4324-4341.

Lu K, Li T, He J, Chang W, Zhang R, Liu M, Yu M, Fan Y, Ma J, Sun W, et al. 2018. qPrimerDB: a thermodynamics-based gene-specific qPCR primer database for 147 organisms. *Nucleic Acids Research* **46**(D1): D1229-D1236.

MacKinnon DP, Lockwood CM, Hoffman JM, West SG, Sheets V. 2002. A comparison of methods to test mediation and other intervening variable effects. *Psychological Methods* **7**(1): 83-104.

Wang Q, Tao T, Zhang Y-J, Wu W-Q, Li D-W, Yu J-L, Han C-G. 2011. Rice black-streaked dwarf virus P6 self-interacts to form punctate, viroplasm-like structures in the cytoplasm and recruits viroplasm-associated protein P9-1. *Virology Journal* **8**: 24.

Wei H. 2019. Construction of a hierarchical gene regulatory network centered around a transcription factor. *Briefings in Bioinformatics* **20**(3): 1021-1031.

Wei M, Liu Q, Wang Z, Yang J, Li W, Chen Y, Lu H, Nie J, Liu B, Lv K, et al. 2020. PuHox52-mediated hierarchical multilayered gene regulatory network promotes adventitious root formation in Populus ussuriensis. *New Phytologist*.
